# Response retention and apparent motion effect in visual cortex models

**DOI:** 10.1371/journal.pone.0293725

**Published:** 2023-11-02

**Authors:** Vasilii S. Tiselko, Maxim Volgushev, Dirk Jancke, Anton V. Chizhov

**Affiliations:** 1 Laboratory of Complex Networks, Center for Neurophysics and Neuromorphic Technologies, Moscow, Russia; 2 Computational Physics Laboratory, Ioffe Institute, Saint Petersburg, Russia; 3 Department of Psychological Sciences, and the Institute for the Brain and Cognitive Sciences, University of Connecticut, Storrs, CT, United States of America; 4 Optical Imaging Group, Institut für Neuroinformatik, Ruhr University Bochum, Bochum, Germany; 5 MathNeuro Team, Inria Centre at Universite Cote d’Azur, Sophia Antipolis, France; Justus Liebig Universitat Giessen, GERMANY

## Abstract

Apparent motion is a visual illusion in which stationary stimuli, flashing in distinct spatial locations at certain time intervals, are perceived as one stimulus moving between these locations. In the primary visual cortex, apparent-motion stimuli produce smooth spatio-temporal patterns of activity similar to those produced by continuously moving stimuli. An important prerequisite for producing such activity patterns is prolongation of responses to brief stimuli. Indeed, a brief stimulus can evoke in the visual cortex a long response, outlasting the stimulus by hundreds of milliseconds. Here we use firing-rate based models with simple ring structure, and biologically-detailed conductance-based refractory density (CBRD) model with retinotopic space representation to analyze the response retention and the origin of smooth profiles of activity in response to apparent-motion stimuli. We show that the strength of recurrent connectivity is the major factor that endorses neuronal networks with the ability for response retention. The same strengths of recurrent connections mediate the appearance of bump attractor in the ring models. Factors such as synaptic depression, NMDA receptor mediated currents, and conductances regulating spike adaptation influence response retention, but cannot substitute for the weakness of recurrent connections to reproduce response retention in models with weak connectivity. However, the weakness of lateral recurrent connections can be compensated by layering: in multi-layer models even with weaker connections the activity retains due to its feedforward propagation from layer to layer. Using CBRD model with retinotopic space representation we further show that smooth spatio-temporal profiles of activity in response to apparent-motion stimuli are produced in the models expressing response retention, but not in the models that fail to produce response retention. Together, these results demonstrate a link between response retention and the ability of neuronal networks to generate spatio-temporal patterns of activity, which are compatible with perception of apparent motion.

## Introduction

**Fig 1:**
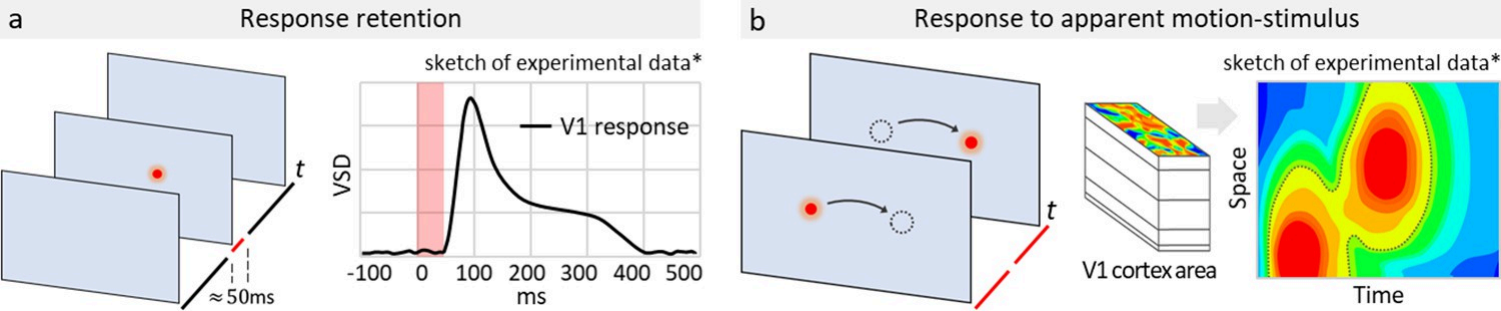
Challenge for the modeling: Response retention and activity patterns mediating apparent motion phenomena in visual cortex. Sketches in a,b show stimulation paradigms and “best approximations” of available experimental observations with voltage-sensitive dyes. (a) Long-lasting response (right panel) to a short (50 ms) stimulus shaped as a small Gaussian blob (left), as in experiment from [1]. (b) An apparent motion stimulus, consisting of a spot of light briefly flashing at two distinct locations (left panel) evokes a continuous activity in space-versus-time coordinates (right). Middle panel in b shows a multi-layer fragment of V1, with the observed surface area.

Experimental evidence shows that responses of neuronal populations in visual cortex to presentation of brief stimuli last considerably longer than the stimulus itself. This effect, which we refer to here as *response retention*, was observed in visual cortex of diverse species, including monkeys [1–3], cats [4], ferrets [5] and mice [6–8]. For example, imaging neuronal activity using voltage-sensitive dyes revealed that in visual cortex of awake monkey response to presentation of a brief 50 ms stimulus lasts for over 300 ms (Fig 1A; [1]). Observed response retention in visual cortex neurons might be a mechanism which mediates the availability of visual information for processing long after a briefly presented stimulus is turned off, and underlie the phenomenon of the persistence of vision, well-described in psychophysics of visual perception [9–12]. Response retention in visual cortex might also be critical for the phenomenon of apparent motion, in which rapid presentation of stimuli at spatially separate locations can produce perception of a continuously moving stimulus (for review see [13–15]; Fig 1B). Indeed, flashing stimuli inducing apparent motion and continuously moving stimuli produce similar spatio-temporal patterns of activity in the visual cortex [4, 16–18].

Despite experimental evidence for response retention and its possible relevance for multiple aspects of visual perception, mechanisms mediating response retention escape mechanistic understanding. Reproducing response retention in computer models is a challenge, because it requires to explain the origin of slow processes taking place in the system built of fast components. Electric processes in single neurons are fast, with kinetics of the slowest components such as NMDA receptor mediated currents not exceeding 150 ms, which is not sufficient for explaining longer-lasting response retention. Hence, response retention must be a network phenomenon, determined by the properties of neuronal connectivity that should be able to generate reverberating activity, thus allowing to preserve and process information about stimuli over extended time periods.

Here we set to reproduce response retention in models consisting of interconnected neuronal populations and determine the contribution of diverse properties of neurons and neuronal connectivity to response retention. We start with the «minimal» firing-rate model with a ring structure that is capable of reproducing the response retention ("Ring model", [19, 20]) and then analyze a ring-structured firing-rate based model which includes synaptic depression and slow kinetics of NMDA receptors (ring-based model with synaptic depression and NMDA receptors, SDN model, [21]). Using these models, allowing for analytical solutions and systematic investigation of parameter space using numeric simulations, we determine critical factors contributing to response retention. Specifically, we describe the role of the recurrent connectivity and its strength, synaptic depression, synaptic currents with slow and fast kinetics, and of the propagation of activity through the multiple layers of multi-layer model networks. Next, we validate results obtained with the ring-structured models and extend the analysis of response retention using a complex biologically detailed model with retinotopic representation of space, the conductance-based refractory density (CBRD) model [22]. Finally, we demonstrate that CBRD model reproducing the response retention can also produce smooth spatio-temporal patterns of activity in response to apparent-motion stimuli, as reported in experimental studies.

## Materials and methods

### Firing-rate ring model

**Fig 2:**
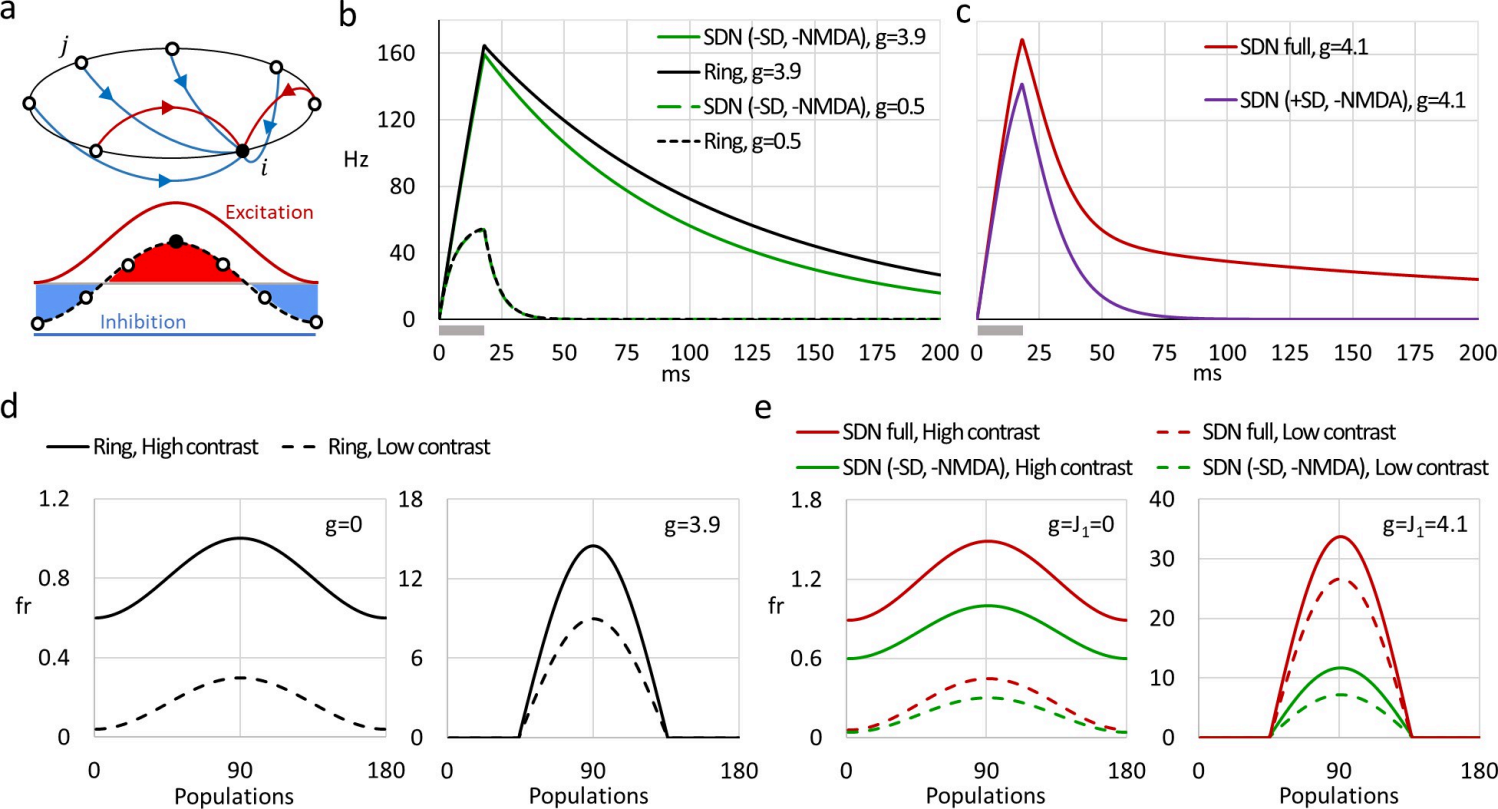
Response retention in ring-structured models. (а) Structure scheme of a ring model. For one node, dominating recurrent connections are shown, tuned excitatory (redlines) and broad inhibitory (bluelines) (bi-directional; arrows indicate only one direction for clarity), and resulting excitation/inhibition connectivity profile (lower panel) are shown. (b) Firing rate response to a flash-stimulus (18 ms, grey horizontal bar below X-axes) in the Ring model (black traces) and SDN model without synaptic depression and NMDA currents (green traces, SDN (-SD,-NMDA)) with either strong (solid lines) or weak (dashed lines) recurrent connections. (c) Responses of the SDN models, full (SDN full, red trace) and without the NMDA receptors (SDN (+SD,-NMDA), violet trace) to a 18 ms flash-stimulus. (d-e) Contrast invariance of orientation tuning in ring-structured models requires strong recurrent connectivity. Orientation tuning of responses to oriented bar stimuli of high or low contrast, (d) in the Ring model with no recurrent connections (g = 0) or with strong connections (g = 3.9); and (с) in the full SDN model (red traces) and SDN model without synaptic depression and NMDA receptors (SDN (-SD, -NMDA), green traces).

The Ring model, originally introduced to describe the activity of an orientation hypercolumn of the primary visual cortex, consists of populations of excitatory and inhibitory neurons that are evenly distributed along the ring according to the preferred orientation of a stimulus [19, 20, 23], (see Fig 2A). The spiking activity of inhibitory neurons in this model is recursively proportional to the activity of neighboring excitatory neurons. The firing-rate model is described by a system of first order differential equations, in which the dependence of the firing rate on synaptic current is represented by a threshold-linear function:

$$\tau_{r}\frac{df_{i}}{dt}=-f_{i}+f^{st}(I_{i}^{syn}),$$ (1)

where *τ*_*r*_ is the time constant, *f*_*i*_ is the firing rate of *i*-population, *f*^*st*^(*x*) = [*x*]_+_. $I_{i}^{syn}$ is synaptic current of *i*-population described as follows:

$$I_{i}^{syn}=I_{0}+I_{1}\;\cos(\theta_{i}-\theta_{0})+N^{-1}\sum_{j=1}^{N}(J_{0}+J_{1}\;\cos(\theta_{i}-\theta_{j}))f_{j},$$ (2)

where *I*_0_ is current produced by the homogeneous part of a stimulus, the second term (*I*_1_) is current produced by the oriented part of the stimulus with the orientation *θ*_0_. *θ*_*i*_ = 2*πi*/*N* is the angle of the preferred stimulus orientation. The last term is synaptic current generated by the recurrent connections, *J*_0_, *J*_1_ are the connection strength parameters. *N* is the number of populations in the hypercolumn. Numerical solutions were calculated for a model with 300 nodes.

### Ring model with Synaptic Depression and NMDA currents (SDN)

The SDN model has same structure as the Ring model, but describes activity of neuronal populations in terms of the dynamics of the current, instead of the firing rate. The SDN model includes synaptic depression, NMDA channels with slow kinetics, and recurrent connections [21]. The basic system of differential equations describes the dynamics of the current as follows:

$$\tau \frac{dI^{i}(t)}{dt}=-I^{i}(t)+I_{stim}^{i}+I_{lat}^{i}(t)+I_{ff}^{i}(t),$$ (3)

where *τ* is the time constant of excitatory transmission, $I_{stim}^{i}=A{\left[\cos\left(2\pi (i-k)/N\right)\right]}_{+}$ is current produced by the stimulus, $I_{lat}^{i}(t)=\sum_{j}w_{ij}^{lat}P_{rel}^{j}(t)r^{j}(t)$ is the input current from lateral connections, with the matrix of lateral connections $w_{ij}^{lat}=g_{lat}(1-\delta_{ij})\cos\left(2\pi (i-j)/N\right)$ and their strength *g*_*lat*_. $I_{ff}^{i}(t)=\sum_{j}w_{ij}^{ff}P_{rel}^{j}(t)r^{j}(t)$ is the input current from layer-to-layer, feed-forward connections, where $w_{ik}^{ff}=g_{ff}\cos\left(2\pi (i-k)/N\right)$ is the matrix of these connections, and *g*_*ff*_ their strength.

The synaptic depression affects the current through the release probability *P*_*rel*_(*t*) according to the first order differential equation:

$$\tau_{depr}\frac{dP_{rel}(t)}{dt}=P_{0}-[1+\tau_{depr}r(t)(1-f)]P_{rel}(t),$$ (4)

where *τ*_*depr*_ = 500*ms* is time constant of recovery of a synapse toward its default release probability *P*_0_ = 1. The depression factor *f* = 0.8 describes how much the synapses depress after each spike, thus changing the release probability (*P*_*rel*_→*fP*_*rel*_). The firing rate is determined by a simple threshold function equivalent to that in the Ring model, but calculated depending on the sum of fast and slow excitatory processes $r(t)={\left[I^{fast}(t)+I^{slow}(t)\right]}_{+}$, where *I*^*fast*^ corresponds to currents that provide fast synaptic interaction with the time constant in Eq (3) as *τ* = *τ*^*fast*^ = 5*ms*, and *I*^*slow*^ corresponds to slow NMDA receptor currents with *τ* = *τ*^*slow*^ = 150*ms*. Numerical solutions were calculated with 300 nodes.

To summarize, differences of the SDN model from the Ring-model include: current-based description (Eq 3), addition of synaptic currents with fast and slow dynamics (AMPA and NMDA), and addition of synaptic depression. In their core, however, the models are similar in that they share same structure, and dynamics of their activity is determined by the strength of recurrent connectivity. In the SDN model without NMDA currents and synaptic depression, and connectivity parameters corresponding to those in the Ring model (*J*_1_~*g*_*lat*_, *J*_0_ = 0,), response dynamics in the two models is essentially the same (see Results for detail). The code for the ring models, written in Delphi-Pascal language, is available upon request.

### CBRD model (see the S1 Methods for a detailed description)

The biologically-detailed CBRD (conductance-based refractory density) model describes excitatory and inhibitory neuronal populations of the primary visual cortex (V1) receiving retinotopically-organized input from the lateral geniculate nucleus of the thalamus (LGN), which in turn receives input from the retina [22, 24]. Activity of LGN neurons is calculated using a filter-based model of receptive fields and firing at the retinal inputs. The spatial filters have center-surround structure determined by axonal projections of the retinal ganglion cells described as a difference of two axisymmetric Gaussian functions. Input from the LGN to the cortex is calculated using spatio-temporal convolution of the LGN activity with the elongated profile of LGN projections to the cortex, where this elongation determines the origin of orientation preferences for cortical neurons. The convolution determines the spatial mapping between the populations of thalamic and V1 neurons, takes into account different response kinetics of thalamic neurons, and provides basic features for orientation and direction selectivity of V1 neurons. It thus determines the orientation selectivity map of V1, with systematic changes of preferred orientations over the cortical surface and pinwheels structure (Fig 5C). The equations for the calculation of the thalamic footprints on the cortex are described in the section "Thalamic input to V1" of the S1 Methods, and are discussed in [21].

Visual cortex is modelled as a two-layer structure mimicking layers 4 and 2/3 of V1. Layer 4 receives LGN input and provides feed-forward input to V1. Populations of excitatory (E) and inhibitory (I) neurons in each layer are connected with recurrent connections within and between layers (see Fig 5A). Neuronal populations of two-compartment Hodgkin-Huxley-like neurons are modelled using the conductance-based refractory density approach [25], with which instantaneous population firing rates are calculated. Each population receives input signals from other populations in form of synaptic conductances (AMPA, NMDA, GABA). Connections express synaptic depression. The output signal of a population is calculated by convolving its firing rate with the profile of its projections. The horizontal cortical connectivity is described by Gaussians with connectivity profiles of E-to-I and I-to-E broader than the profiles of E-to-E and I-to-I connections. Further description of CBRD model is provided in Results (Fig 5 and related text), and complete description is provided in the S1 Methods and in [22]. The code for the CBRD model, written in Delphi-Pascal language, is available upon request.

## Results

To determine parameters of neuronal electrophysiology, synaptic transmission and connectivity which are critical for retention of responses to brief visual stimuli we first use models with a simple ring-structure, because such models allow for analytical solutions and systematic investigation of parameter space using numeric simulations.

### Models with ring-structure

#### Ring model

A firing rate model with a simple ring structure (*Ring model*) was proposed to describe activity in orientation hypercolumns of the primary visual cortex, origin of pinwheels in orientation preference maps and contrast invariance of orientation tuning [19, 20, 23]. In the ring model, each orientation column is represented by a node in which neurons are tuned to stimuli of a certain orientation. Nodes are arranged in a ring covering all orientations, thus representing a hypercolumn, and are connected by recurrent connections. Each node is connected to other nodes, with cosine–distributed weights of excitatory connections, and equally weighted inhibitory connections (Fig 2A). For simulations described in this paper, we used a ring model with 300 nodes, which is well within the plateau of the dependence of simulation results on the number of nodes.

**Fig 3:**
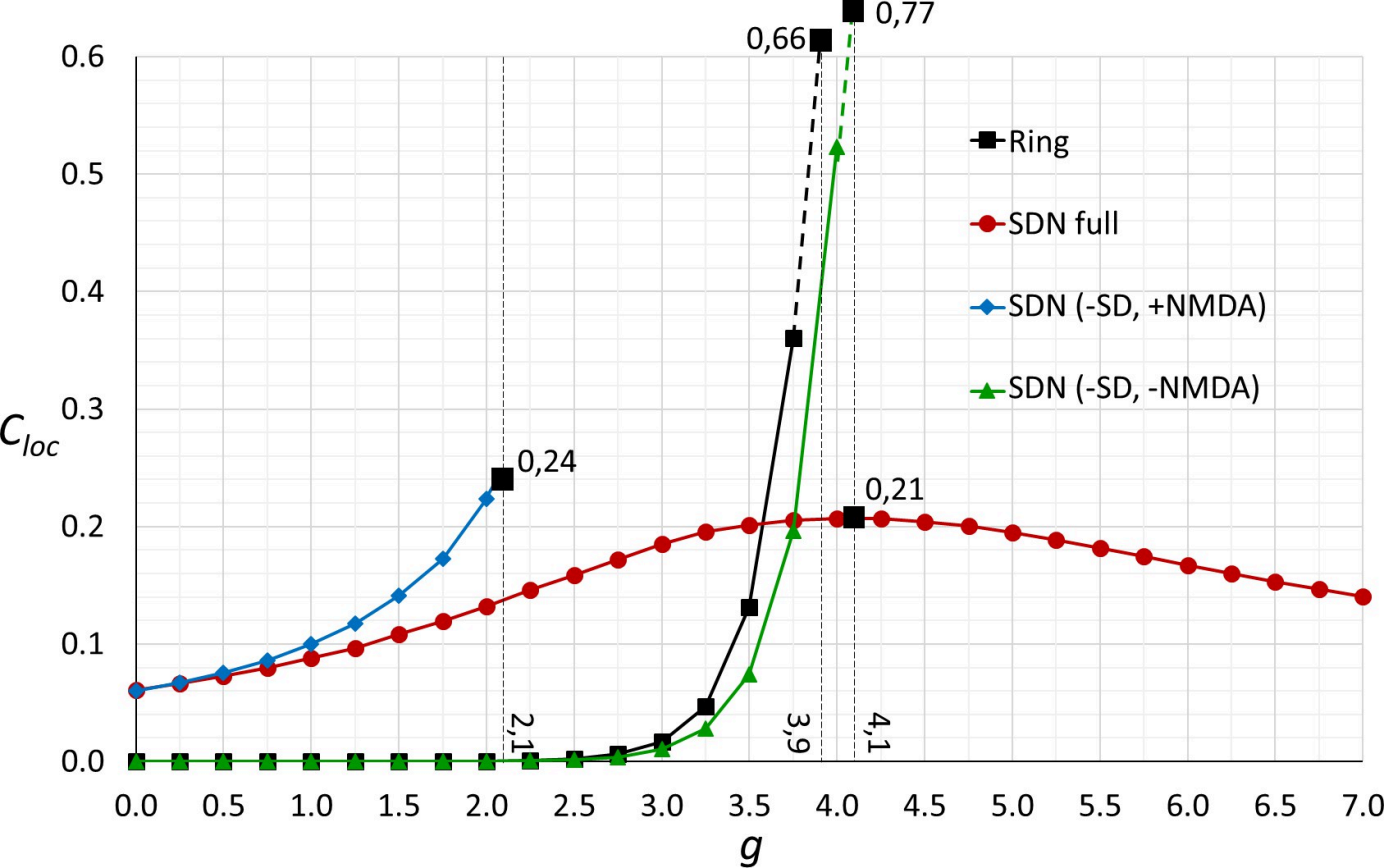
Dependence of response retention on the strength of recurrent connections in ring-structured models. The retention ratio *C*_*loc*_ (ratio of response amplitude 100 ms after the peak to the peak amplitude) is plotted against the strength of recurrent connections for four models with ring structure: the Ring model (black, squares); the complete SDN model (red, circles); the SDN model without synaptic depression (blue, diamonds); the SDN model without both synaptic depression and NMDA receptor currents (green, triangles). Dotted lines indicate the upper boundaries of connection strength at which activity in the models becomes unstable (or maximal *C*_*loc*_ is reached in the complete SDN model). Numbers next to X-axis indicate boundary values of recurrent connection strength; numbers at the intersection of the boundaries with data fits indicate retention ratio *C*_*loc*_ for respective models.

In response to brief stimulus presentation (18 ms), a single node of the ring model with weak recurrent connections generates a transient response, with activity decaying at a rate determined by the time constant in the firing rate equation (Methods, Eq 1), and returning to zero within few milliseconds after the stimulus offset (Fig 2B, black dashed line). In contrast, response of the ring model with strong recurrent connections decays slowly, with the duration of response at half-peak amplitude reaching 70 ms, and firing rate remaining at a substantial level even 200 ms after the stimulus (Fig 2B, black solid line). The duration of the response is thus comparable to the experimentally observed (Fig 1). To quantify the response retention, we used the retention ratio *C*_*loc*_ calculated as the ratio of response amplitude 100 ms after the peak to the peak amplitude (Eq 45 in S1 Methods). This measure is normalized between 0 and 1, thus facilitating comparison between different models with intrinsically different response kinetics and between different stimulation paradigms. Note that 100 ms is longer than the duration of any stimuli considered here as short, and longer than the time scales of responses in the LGN and of monosynaptic cortical responses. For the response of the ring model with strong connections (from Fig 2B) the retention ratio *C*_*loc*_ = 0.37. Analysis of responses of the ring model with systematically increasing strength of recurrent connections *g* revealed that response retention is absent (*C*_*loc*_ = 0) in the models with *g* <2.5. Response duration starts to exceed 100 ms (*C*_*loc*_ > 0) with *g*≈3. Response retention becomes pronounced (*C*_*loc*_ > 0.1) with *g*≈3.5, and steeply increases with further increase of connection strength (Fig 3, black squares and black line). At a critical value of connectivity strength *g* = 3.9, the model becomes unstable, as it was reported in the original study [20]. In their original study of the ring model Hansel and colleagues [20] reported that strong recurrent connectivity brings about one further experimentally observed feature of visual cortex neurons, contrast invariance of orientation tuning. Indeed, in the ring model with weak or absent recurrent connections, increasing stimulus contrast leads to an increase of responses to all orientations, and thus decrease of the selectivity (Fig 2D, left plot). In the model with strong connectivity, orientation tuning at any contrast is sharper than in the model with weak connections, and it remains sharp independently of the stimulus contrast (Fig 2D, right plot). Thus, strong recurrent connectivity provides the ring model both with contrast-invariance of orientation tuning and appearance of a bump attractor which shape is invariant for a broad range of the amplitude of input, as described before [20], as well as with the ability to generate prolonged activity in response to brief stimuli–response retention.

#### SDN model

The firing rate ring model considered above does not take into consideration some important factors shaping neuronal response dynamics, such as kinetics of synaptic currents and synaptic dynamics. To analyze the contribution of these factors to response retention, we next used a model proposed by van Rossum and colleagues [21], which includes synaptic depression with time constant *τ* = 500 ms and NMDA currents with time constant *τ*_*NMDA*_ = 150 *ms* (*SDN model*). SDN model is a firing rate-based model, in which the total current determines the firing rate according to a threshold function. In our simulations we used SDN model with the same structure as the ring model, with 300 nodes connected by recurrent excitatory and inhibitory connections (see Methods).

To verify that results of the above analysis are applicable to the SDN model we first used a reduced SDN model with blocked NMDA currents and inactivated synaptic depression. With these settings, the SDN model closely reproduces the results obtained with the firing-rate ring model. Responses to a brief stimulus presentation are transient in the reduced SDN model with weak connections (Fig 2B, green dotted line), but are prolonged and express clear response retention (retention ratio *C*_*loc*_ = 0.29) in the model with strong connectivity (Fig 2B, green solid line). Dependence of the response retention ratio on the strength of recurrent connections in the reduced SDN model is also very similar to that of the ring model (Fig 3, compare green triangle symbols and green line to black squares and black line).

Furthermore, similarly to the ring model, contrast invariance of orientation tuning in the reduced SDN model also requires strong connectivity (Fig 2E, green lines). Thus, the SDN model without NMDA currents and synaptic depression has same properties as the ring model. This allows us to use SDN model for analysis of the effects of NMDA currents and synaptic depression on response retention.

In the complete SDN model with strong connections response to a brief stimulus is prolonged (Fig 2C, red trace), though its decay dynamics is different from the response decay in the ring model (Fig 2, compare red trace in b to the red trace in c). In the SDN model, response decays faster during the first ~50 ms after the offset of the stimulus, but then the decay becomes very slow so that even at 200 ms the firing rate remains at ~20% of the peak amplitude (Fig 2C, red trace). Response dynamics in the full SDN model shows a closer correspondence to experimentally observed response in the visual cortex (Fig 1), than the dynamics of the ring model response. Analysis of the dependence of response duration on the strength of connectivity shows that, in the complete SDN model, responses to brief stimuli are prolonged even with weak connectivity (Fig 3, red circles), apparently due to the slow kinetics of NMDA currents (*τ*_*NMDA*_ = 150 *ms*). With increasing the strength of connectivity response retention progressively increases and retention ratio reaches values of *C*_*loc*_ > 0.2 in the range of connection strengths 3.5 < *g* < 4.5, with the maximum *C*_*loc*_ = 0.21 at *g* = 4.1. With further increase of connection strength, the retention ratio decreases to *C*_*loc*_ = 0.14 at *g* = 7 (Fig 3, green circles).

Synaptic depression and NMDA currents affect response retention in SDN model in the opposite ways. Blocking NMDA currents leads to the loss of response retention even in SDN model with strong connectivity (Fig 2C, violet trace). Response retention is expressed in the full SDN model, and in the reduced SDN model without depression and NMDA currents, but is abolished in the model with depression but without NMDA currents. Thus, synaptic depression effectively prevents continuing recurrent excitation and response retention, but NMDA currents counteract this effect of synaptic depression on response duration. Removing synaptic depression from the model leads to a steeper increase of response retention ratio than in the full SDN model (Fig 3, compare blue diamonds to red circles), and with connection strength coefficient *g* = 2.1 the model without synaptic depression becomes unstable.

In the complete SDN model, contrast invariance of orientation tuning critically depends on the presence of strong recurrent connections, as it does in the ring model. Orientation tuning is contrast invariant in SDN model with strong connections (Fig 2E, right plot), but not in the model with weak or absent connections (Fig 2E, left plot). Thus, complete SDN model with strong connections reproduces both the response retention and contrast invariance of orientation tuning.

#### Multi-layer SDN model

**Fig 4:**
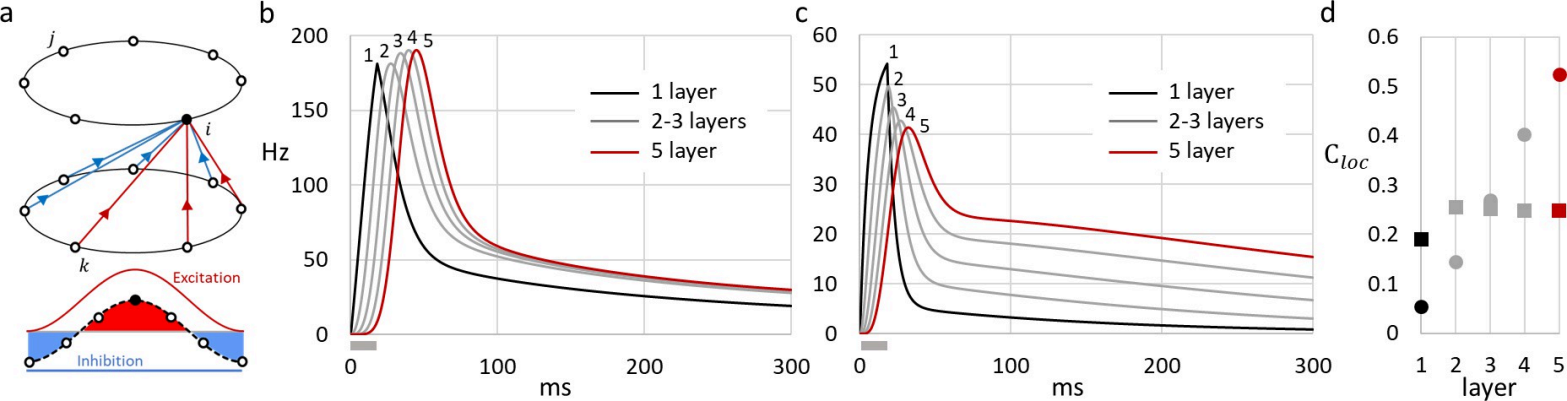
Response retention increases in multi-layer models. (a) Structure scheme of a multi-layer SDN model. Only connections between layers for one recipient are shown, where color of the arrows indicates whether excitation (red) or inhibition (blue) dominates at the location. Internal connections within each layer are the same as shown in Fig 2A; (b-c) Firing rate responses to a flash stimulus (18 ms; grey horizontal bar below X-axes) in five layers of multi-layer SDN models with strong connections within each layer (b) or weak within-layer connections (c). (d) The retention ratio *C*_*loc*_ plotted against layer for models with strong (squares, data from b) and weak (circles, data from c) connections within layer. Note that in the model with strong connections response retention is present already in the first layer, improves in the second layer and then levels-off. In contrast, in the model with weak connections, retention is not present in the first layer, but develops and progressively increases as activity spreads across the layers.

Next, we analyzed response retention in multi-layer models, because most of brain regions including the visual cortex have multi-layer structure. Each layer of the model was represented by a complete SDN model as described above. The layers were connected by feedforward connections. Each node of one layer sent excitatory connections to the nodes of the next layer with cosine-distributed weights, and inhibitory connections of equal weight to all nodes of the next layer (Fig 4A; see Methods for detail).

We consider only feedforward connections between the layers because introducing recurrent connections between layers would be equivalent to increasing the number of nodes within one layer. The structure of the multi-layer model used in our simulations is equivalent to the structure of the multi-layer CSD model introduced by van Rossum and colleagues [21] (see Methods, section SDN model).

Fig 4 shows responses of excitatory neurons from each of the five layers of the multi-layer SDN model. In the model with strong connectivity within each layer, response retention is apparent already in the first layer (Fig 4B, black line, *C*_*loc*_ = 0.19). Response retention becomes even more pronounced in the second layer (*C*_*loc*_ = 0.26), but then saturates and remains at the same level in the subsequent layers 3–5 (Fig 4B, grey and red traces; and Fig 4D, squares). In contrast, in the model with weak within-layer connections responses change markedly with each consequent layer. In the first layer the response is essentially transient, with negligible late component (Fig 4C, black solid line, *C*_*loc*_ = 0.05). With propagation of activity through each subsequent layer, peak amplitude of the response decreases but its lasting component increases, leading to a progressive increase of the retention ratio which reaches the value of *C*_*loc*_ = 0.52 in the layer 5 (Fig 4C, grey and red traces; and Fig 4D, circles). Note that response retention in layers 4 and 5 of the multi-layer model with weak within-layer connectivity is more pronounced, with higher values of the retention ratio, than in any layer of the model with strong within-layer connections (Fig 4D), or in one-layer SDN model with any strength of recurrent connectivity (Fig 3, red circle symbols and line). Thus, response retention is enhanced in multi-layer structures with weak (or moderate) within-layer connectivity.

To summarize, results of simulations with ring-structured models show that response retention can be brought about within a single layer by strong recurrent connectivity, or at higher layers of multi-layer models even with weak within-layer connections. Within a single layer, response retention is hindered by synaptic depression but is promoted by NMDA currents.

### Conductance-based refractory density (CBRD) model

We used a biologically detailed conductance-based refractory density (CBRD) model with retinotopic structure to validate the above results, analyze the contribution of additional biophysical processes to response retention, and extend our analysis to spatio-temporal profiles of activity, allowing to relate response retention to generation of activity profiles produced by apparent-motion stimuli.

**Fig 5:**
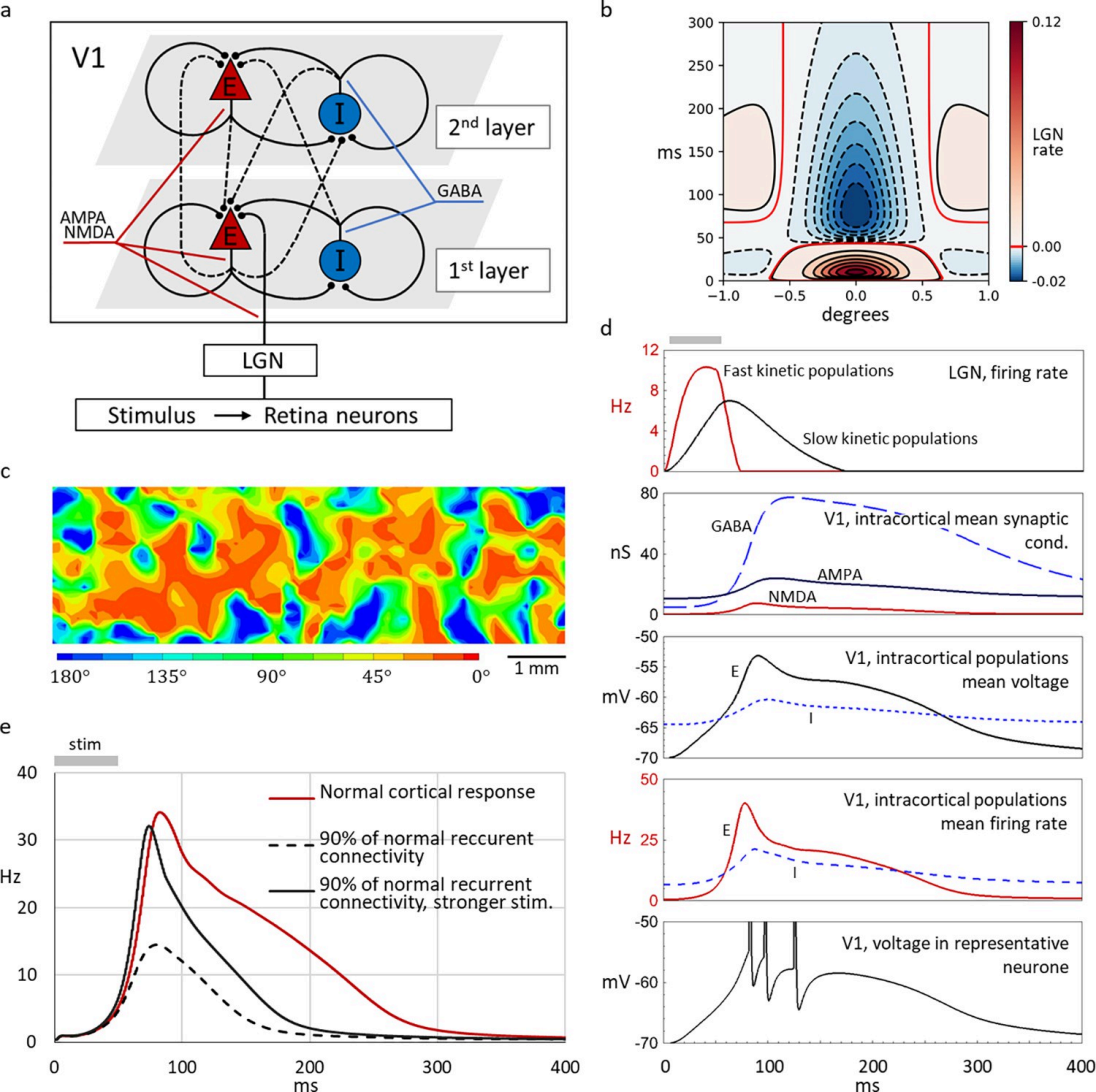
Biophysically detailed conductance-based refractory density (CBRD) model. (a) Structure scheme of the CBRD model. Input from the thalamus (LGN) arrives at excitatory neurons in the first layer of the visual cortex (V1). Connections between excitatory and inhibitory neurons within each of the two cortical layers are shown with solid lines; connections between the layers are shown with dashed lines. Excitatory synapses contain AMPA and NMDA receptors, inhibitory synapses contain GABA receptors. (b) Spatio-temporal profile of the receptive field of LGN neurons, plotted using analytically calculated response to a delta function stimulus. Red isolines show the boundaries between inhibitory and excitatory zones. (c) The map of orientation bias of the thalamocortical input to neurons in the simulated V1 region, based on data from [26]. (d) Components of the model’s response to a brief presentation of a light spot (1^O^ diameter, 50 ms; grey bar above the top panel): the firing rate of LGN neurons; the mean AMPA, NMDA and GABA synaptic conductance in excitatory cortical neurons (assuming the membrane area to be 0.7∙10^−5^ *cm*^2^, as in Methods); the mean membrane potential of excitatory and inhibitory populations of cortical neurons; the mean firing rate of excitatory and inhibitory populations of cortical neurons; the membrane potential trace in a representative cortical neuron. (e) Firing rate response in excitatory cortical neurons in the model with standard settings (red trace), with the strength of recurrent connections reduced to 90% (black dashed line) and in the model with reduced recurrent connectivity but in response to a stronger stimulus (black solid line). Note that the duration of cortical response is reduced in the model with reduced recurrent connectivity, and that increasing the stimulus strength increases the amplitude but not the duration of response.

The CBRD model is based on previous work [22, 24], and describes neuronal populations of the primary visual cortex (V1) receiving retinotopically-organized input from neurons in the lateral geniculate nucleus of the thalamus (LGN), which in turn receive input from neurons in the retina activated by external visual stimuli (Fig 5A). In the visual cortex the model includes populations of two-compartment Hodgkin-Huxley-type excitatory and inhibitory neurons organized in two layers mimicking layers 4 and 2/3 of V1 (the size of the simulated cortical region is about 8 mm long and 3 mm wide). Within each layer, neurons are connected by recurrent connections mediated by synapses with AMPA and NMDA or GABA receptors. Synaptic connections in each neuronal population have log-normal distribution of synaptic weights and express synaptic dynamics. The second layer of the CBRD model has the same structure as the first layer, but receives no LGN input. Populations of excitatory and inhibitory neurons of the second layer are reciprocally connected to excitatory neurons from the first layer. Excitatory neurons in the second layer also receive inputs from inhibitory cells from the first layer (Fig 5A, see Methods section for full description of neurons and connectivity). Neurons in the LGN have spatially-localized receptive fields with distinct temporal dynamics of activity (Fig 5B). Neurons in the visual cortex are tuned to different orientations, building on the visual cortex, in addition to the retinotopic map of space, a systematic representation of preferred orientations. Orientation preference map of V1, obtained in experiments [26], was used in our simulations; it is shown for the simulated domain in Fig 5C.

In our model, for each point in the cortex, the orientation angle for the elongated footprint of LGN projections to cortical neurons was determined using the experimental orientation map [26]. This elongation creates an orientation bias in cortical responses. Since cortical neurons that receive thalamic inputs with similar orientation bias are localized in columns, the local interactions, structured by Gaussian connectivity, lead to intracortical sharpening of the orientation tuning [24, 27]. This sharpening is not only driven by excitation but also by inhibition and voltage-gated mechanisms including shunting. Thus, though the prior orientation bias is set by thalamo-cortical projections, the final orientation tuning emerges from the full model. The CBRD approach, in contrast to alternative neural mass-like models, provides accurate transient dynamics of the sharpening of orientation tuning. The spread of activity within the cortex in response to a small spot stimulus, known as the point-spread function, is characterized by the range of the thalamic cell receptive fields, the extent of the footprint of thalamocortical projections, and the scales of the Gaussian connectivity profiles.

In the model with standard settings, excitatory neurons have an increased activity for about 250 ms in response to a brief 50 ms stimulus presentation, thus demonstrating clear response retention (Fig 5E, red trace, *C*_*loc*_ = 0.47). Fig 5D shows dynamics of the key components of the model’s response. In the LGN, firing rate response closely follows the time course of the stimulus in neurons with fast response kinetics, and outlasts the stimulus by ~100 ms in neurons with slow response kinetics. Input from the LGN activates excitatory neurons in V1 which in turn activate intracortical feedforward and recurrent connections, leading to pronounced increases of the mean synaptic conductance through AMPA, NMDA and GABA channels (Fig 5D, panel V1, intracortical mean synaptic conductance). Increase of synaptic conductance leads to depolarization and increase of the firing rate in excitatory and inhibitory neurons. Activity in excitatory neurons is prevailing the activity in the inhibitory population during the period from ~80 ms to ~250 ms after stimulus onset, but then inhibition resumes its dominance because of the background activity of inhibitory neurons. Membrane potential trace in a representative V1 excitatory neuron illustrates that spikes are generated both around the peak of the population mean of the membrane potential response as well as during the later plateau component of the response (Fig 5D, bottom panel). Under the assumption that voltage-sensitive dye signal measured in experiments correlates with membrane potential changes in the population of most numerous, excitatory neurons [28], time course of the response of the model is in a good agreement with the experimentally measured response (Fig 5D, middle panel and Fig 1).

The long-lasting response in CBRD model was observed with excitatory conductance in the recurrent excitatory connections ${\bar{g}}_{AMPA,E}$ starting from ~0.4 mS/cm^2^ (Fig 5E, red trace). With smaller ${\bar{g}}_{AMPA,E}$ = 0.36 mS/cm^2^ (90% of normal) the response became smaller and shorter (Fig 5E, dashed line). Notably, increasing the stimulus strength to achieve the peak amplitude similar to that of the response in the model with strong recurrent connections, did not lead to the increase of the duration of response in the model with weaker connections (Fig 5E, black solid trace).

**Fig 6:**
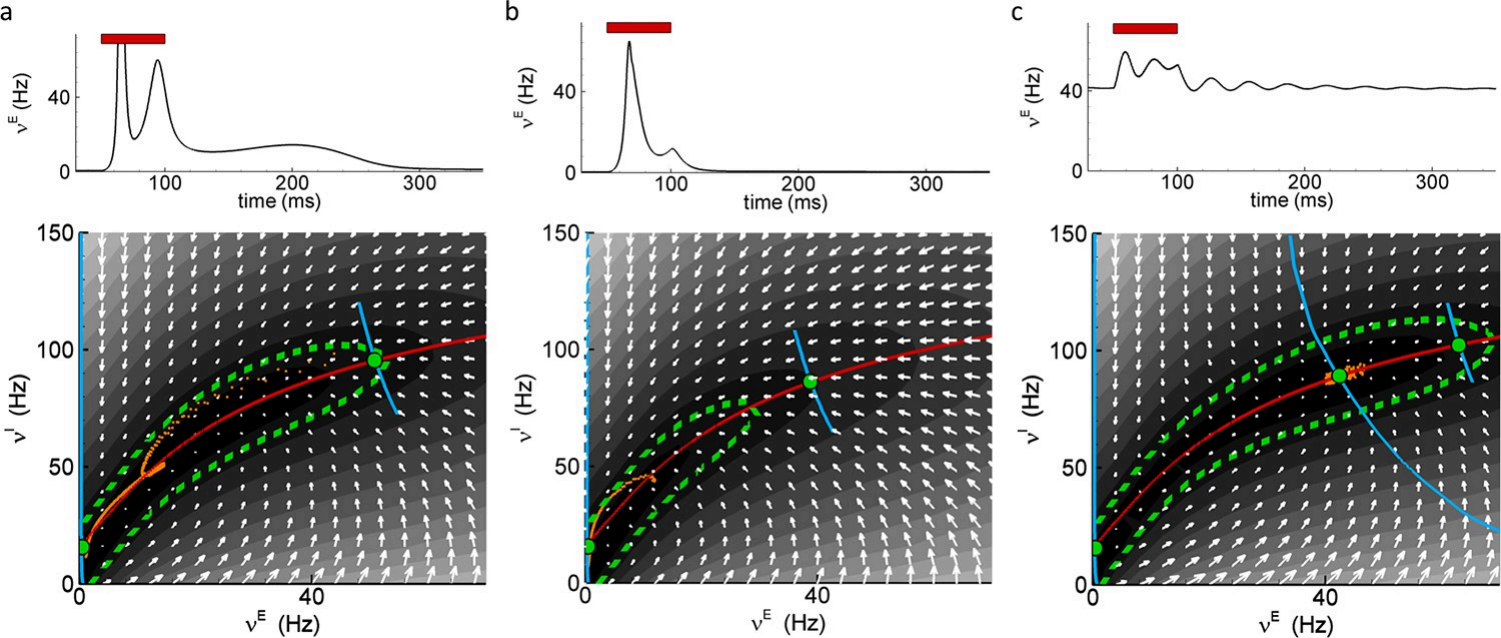
Phase plot analysis of response dynamics in V1 evoked by a step-like change of spatially homogeneous thalamic input *ϕ*_*Th E*_(*x*, *y*, *t*) in CBRD models with different strength of recurrent connectivity. (a-c) Top, the firing rate of excitatory neurons in response to a brief stimulus (50 ms, red horizontal bar). Bottom, vector plots in coordinates of excitatory and inhibitory population firing rates, ν^E^ and ν^I^. The vectors are proportional to dν^E^/dt and dν^I^/dt calculated after holding the model in a fixed (ν^E^, ν^I^)-state. The blue and red curves are the nullclines corresponding to dν^E^/dt = 0 and dν^I^/dt = 0. The long nullclines are for steady-state without stimulation, the short ones are for condition of a constant stimulus. The orange dots mark the trajectory of the response to a short stimulus with a time interval of 0.5 ms after the stimulus offset; green dashed line delineate the region of slow rate changes, ($\sqrt{{\left({d\nu }^{E}/dt\right)}^{2}+{\left({d\nu }^{I}/dt\right)}^{2}}$ < 4 Hz/ms). (a) Model with standard settings, showing response retention. Conductances in recurrent connections were ${\bar{g}}_{AMPA,EE}$ = 0.6 mS/cm^2^ and ${\bar{g}}_{NMDA,EE}$ = 2.4 mS/cm^2^. From high rates at the offset of the stimulus (upper green dot) the system slowly evolves to the resting state (lower green dot). (b) Model with weak recurrent connections, with ${\bar{g}}_{AMPA,EE}$ = 0.4 mS/cm^2^ and ${\bar{g}}_{NMDA,EE}$ = 1.6 mS/cm^2^. The system rapidly evolves from the elevated rates at stimulus offset to the resting state. Response retention is absent in this model. (c) Model with strong recurrent connections with ${\bar{g}}_{AMPA,EE}$ = 0.8 mS/cm^2^ and ${\bar{g}}_{NMDA,EE}$ = 3.2 mS/cm^2^. The system is in high rate state, and rapidly returns to it after stimulation.

The role of recurrent connectivity in response retention in the CBRD model can be understood using phase plot analysis. Fig 6A shows results of such analysis of the CBRD model with standard settings, with the strength of recurrent connections ${\bar{g}}_{AMPA,E}$ = 0.6 mS/cm^2^. Vectors in each point of the phase plot show the direction and the magnitude of changes of the firing rate of excitatory and inhibitory neurons after the rates were fixed until quasi steady-state was reached at respective values (coordinates of the origin of each vector) and the model was then released to follow its own dynamics. Around the nulcline for rate change of inhibitory neurons (red solid line in the vector plot), there is a basin of small vector amplitudes delineated by green dashed line ($\sqrt{{\left({d\nu }^{E}/dt\right)}^{2}+{\left({d\nu }^{I}/dt\right)}^{2}}$ < 4 Hz/ms). Within the basin, gradients for firing rate changes of excitatory and inhibitory neurons are low, and once the rates are within that region, they change only slowly and the model generates prolonged activity.

Response of the model with standard settings to a brief 50 ms stimulus consists of several components (Fig 6A, top panel). The first brief and strong increase of the firing rate in excitatory neurons with the peak at ~230 Hz is followed by a smaller increase of activity with the peak frequency of ~50 Hz and a long decay, lasting up to >200 ms after the stimulus. Trajectory of this second response component is illustrated in the phase plot with orange dots showing the evolution of firing rates of excitatory and inhibitory populations in successive 0.5 ms intervals (Fig 6A, bottom panel). Upon stimulation, the system is shifted towards the high firing rates (right green point), and then gradually evolves to the initial state. As the trajectory enters the low-gradient basin, and especially its lower part (excitatory firing rate <15Hz), changes of activity become very slow as indicated by high density of points on the response trajectory, resulting in a prolongation of response and thus retention of activity.

In the model with a decreased strength of recurrent connections (Fig 6B, ${\bar{g}}_{AMPA,EE}$ = 0.4 mS/cm^2^) the vectors in the phase plot have larger amplitudes, meaning higher gradients for firing rate changes. Steeper slope for the trajectory of rate changes after a perturbation causes a faster relaxation of activity toward the steady state. The basin of low gradients of firing rate changes is smaller and less deep in the model with weak recurrent connections as compared to the model with standard settings (compare phase plots in Fig 6A and 6B). Also, the amplitude of response to the stimulus is smaller in the model with weaker recurrent connections. Consequently, the trajectory of the second component of response to presentation of a brief stimulus is overall shorter, a smaller portion of it goes through the basin of low gradients, and firing rate changes between successive dots are larger. Indeed, the dots composing the response trajectory are less dense than in the standard model. Because of these factors, return of the firing rates to their steady state is fast, and there is no retention of activity in response to a brief stimulus presentation (Fig 6B, upper panel).

In the CBRD model with an increased strength of recurrent connections (Fig 6C, ${\bar{g}}_{AMPA,EE}$ = 0.8 mS/cm^2^) the low-gradient basin in the phase plot is bigger than in the model with standard settings and has a local minimum, and thus a steady state, at high rates (green point at ~40 Hz v^E^). A brief stimulus evokes further increase of firing rates, however, activity in the model does not deviate far from the steady state, and returns back to the high-rate steady state level within <100 ms (Fig 6C). There is no response retention in the model with very strong recurrent connectivity.

Thus, in CBRD model, much like in the models with ring-structure, response retention critically depends on the strength of recurrent connections. The ability to generate prolonged responses to brief stimuli requires that the strength of recurrent connections is within an optimal range. Outside of that range, perturbed activity rapidly returns to the background level, which is close to zero in the model with low or moderate connectivity strength, and at a stable high rate in the model with strong connectivity.

#### Neuron and synaptic properties affecting response retention in CBRD model

**Fig 7:**
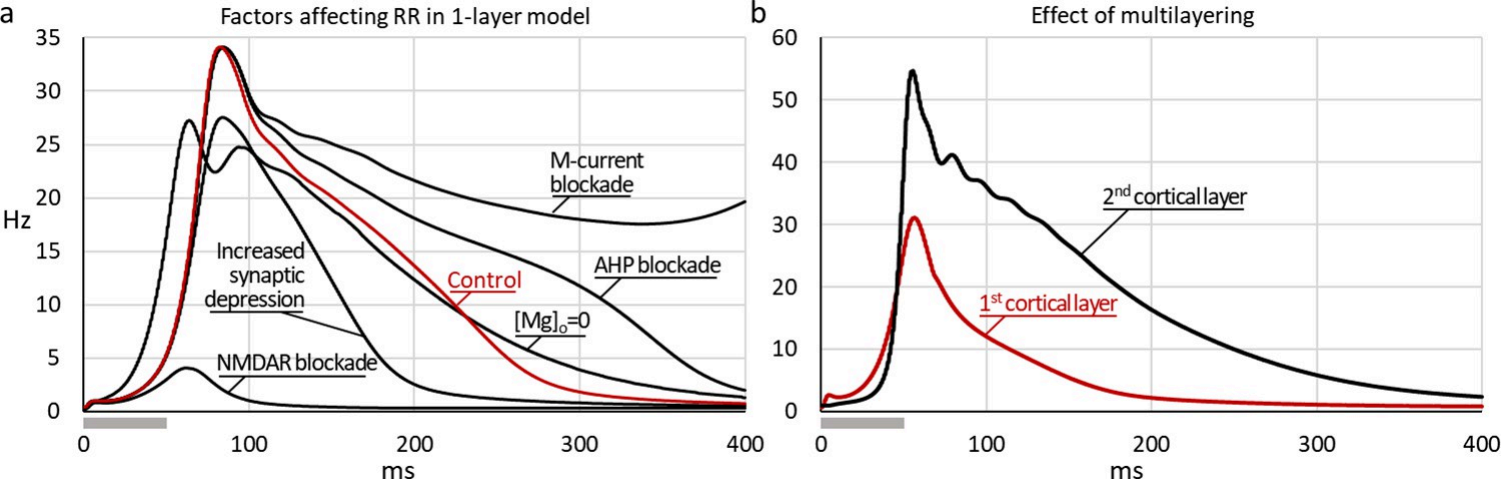
Main factors affecting response retention in the CBRD model. (a) Firing rate responses to a 50 ms flash-stimulus (gray horizontal bar below X-axis) in the single-layer version of CBRD model with standard settings (red trace) and modified parameters, as indicated. Blockade of NMDA receptors leads to a complete loss of response retention (trace NMDAR-blockade), while removing the voltage-dependent magnesium block of NMDARs has little effect on response retention (trace [Mg]_o_ = 0). Increasing the strength of synaptic depression decreases the response retention. In contrast, blockade of conductances contributing to AHP increase the response retention (trace AHP blockade), and blockade of the M-current leads to a sustained response (trace M-current blockade). (b) The responses of two cortical layers to a 50 ms flash-stimulus. Response retention is absent in the first layer (red trace) because of weak within-layer recurrent connections (${\bar{g}}_{AMPA,EE}$ = 0.3 mS/cm^2^), but occurs in the second layer (black trace).

We explored which features of individual neurons and synapses in the CBRD model affect response retention by comparing responses in models with modified parameters to the response of the model with standard settings (Fig 7A, red trace; retention ratio *C*_*loc*_ = 0.47). Strengthening synaptic depression led to a moderate decrease of the peak response amplitude (27 Hz, compared to 34 Hz in control), and to a strong decrease of the response retention (*C*_*loc*_ = 0.16; Fig 7A, trace Increased synaptic depression). Blockade of NMDA receptors led to a yet more dramatic decrease of the response, with peak amplitude of only 4 Hz, and complete loss of response retention (*C*_*loc*_ = 0.10; Fig 7A, trace NMDAR blockade). Notably, voltage-dependent nonlinearity of the NMDA receptor conductance was not essential for response retention. In the model with zero extracellular magnesium concentration ([Mg]_o_ = 0) and reduced strength of NMDA conductance to prevent excessive activity and depolarization block, response retention was still observed and even became slightly stronger than in control (*C*_*loc*_ = 0.62; Fig 7A, trace [Mg]_o_ = 0). In simulations with [Mg]_o_ = 0, the response also starts earlier because the NMDA-channels open already at resting membrane potential, rather than after activation of AMPA receptors had produced an initial depolarization needed to relieve magnesium block of NMDA receptors in nonzero [Mg]_o_.

Blockade of potassium currents enhances response retention. Blockade of the AHP-mediating current prolongs the response (Fig 7A, trace AHP blockade, *C*_*loc*_ = 0.58). With blockade of the M-current the response is also prolonged and the model enters an oscillatory mode after the stimulation, with several waves of activity after the stimulus offset (Fig 7A, trace M-current blockade, *C*_*loc*_ = 0.67).

Because in the ring-structured models response retention could build up as activity propagates through multiple layers (see Fig 4), we asked if in the CBRD model activity evoked by a brief stimulus could persist longer in the second layer as compared to the first layer. (Note that the interlayer connections are one-way, so the activity in the first layer is equivalent to that in the single-layer version of the model.) In the simulation for Fig 7B, the strength of recurrent connections was reduced (${\bar{g}}_{AMPA,EE}$ = 0.3) so that response of neurons in the first layer expressed only moderate retention (Fig 7B, red trace, *C*_*loc*_ = 0.16). In the second layer of that model, response was significantly longer, with higher retention ratio (Fig 7B, black trace, *C*_*loc*_ = 0.47).

Thus, analysis of the CBRD model validated major results obtained with ring-structured models, such as the critical role of recurrent connectivity for response retention, its facilitation by NMDA receptors and suppression by synaptic depression, and enhancement of response retention with propagation of activity through multiple neuronal layers. Analysis of the CBRD model also extends these results revealing that potassium currents (AHP and M-current) limit the duration of response and retention of activity.

### Activity in the CBRD model evoked by apparent motion stimuli

Presentation of flashing stimuli at spatially displaced locations may lead to perception of apparent motion, depending on the timing of stimulation (for review see [13–15]). Imaging using voltage-sensitive dyes revealed that apparent motion stimuli evoke in visual cortex of cats, ferrets and monkeys spatio-temporal patterns of activity that are very similar to those evoked by continuously moving stimuli [4, 5, 16]. Retinotopic representation of space in the CBRD model allows us to simulate the spread of activity in response to stimuli either continuously moving or flashing at spatially displaced locations, and thus to study spatio-temporal patterns of activity that may underlay perception of apparent motion. To compare simulated responses with experimental results, we quantify cortical activity as averaged changes of the membrane potential of excitatory neurons, which is a presumed correlate of the signal measured with voltage-sensitive dyes [28].

**Fig 8:**
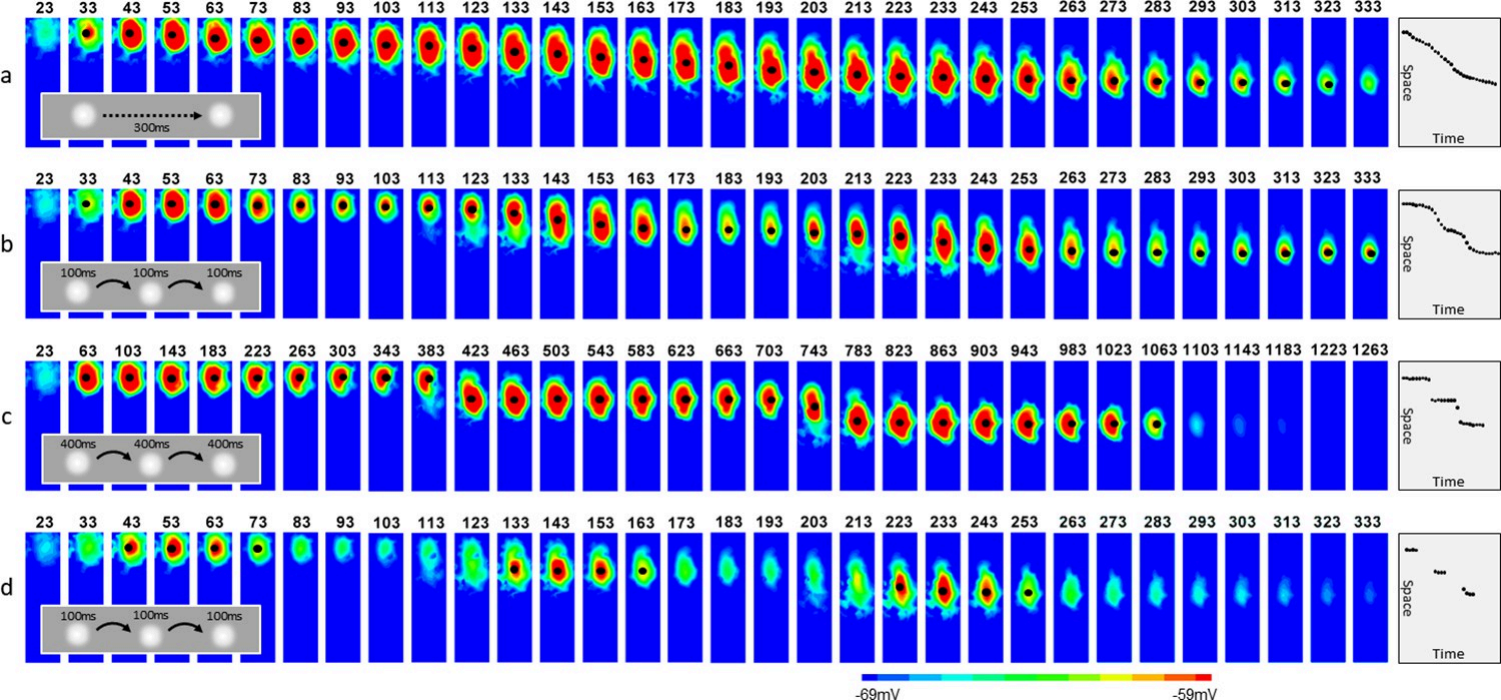
Spatial profiles of activity evoked in CBRD model by either a moving stimulus or patterns of flashing stimuli that may produce apparent motion effect (8.8 mm long region of the simulated visual cortex is shown, the same data as for Fig 9). (a-d) Consecutive frames of spatial profiles of activity in CBRD model evoked by a 1° spot of light, either moving continuously over a distance of 2° (a) or flashing at 3 locations separated by 1°. Black dots in the cortical response heatmaps and additional right panels mark peaks in areas of high activity; the dots are present only in those frames in which the peak activity is above -60mV (red area). The dots reveal the spatio-temporal patterns, which, in panels a and b, are close to straight lines. (b-d). Activity is measured as mean membrane potential of cortical neurons, which provides an estimate of the signal measured in experiments using voltage-sensitive dyes. Timing in ms is indicated above each frame. Insets show schematics of stimulation patterns. Flashing stimuli at short intervals (b, 100 ms) produce activity pattern that closely resembles the response to a moving spot, with a gradual shift of activity (a). Increasing the interval between stimuli to 400 ms disrupts the smooth shift of activity (c, note coarse time scale). Smooth shift of activity is also disrupted in a model with weaker recurrent connectivity (d). See Fig 9 for further analysis.

A moving spot stimulus (1° diameter, moving over a distance of 2°) evokes in the model cortex activity with the focus smoothly shifting with the changing position of the moving spot (Fig 8A). An apparent motion stimulation, flashing the same spot stimulus for 100 ms in succession at three locations separated by 1°, evokes activity that also propagates smoothly between the locations (Fig 8B, frames from 113 to 283 ms). This pattern of activity closely resembles the pattern evoked by a continuously moving stimulus, and might be compatible with perception of apparent motion (compare right panels in Fig 8A and 8B). When the duration of each flashing stimulus is increased to 400 ms, activity becomes contained to each of the three locations, with clearly step-wise transitions of the active region from one location of the flashed stimulus to the next location (Fig 8C). Similarly, clear step-wise transitions of activity focus between sequential stimulated locations is observed in the model with weaker recurrent connections (${\bar{g}}_{AMPA,EE}$ = 0.3) in response to rapidly presented stimuli (100 ms each). As described above, the model with weak recurrent connections does not generate prolonged activity in response to brief stimuli and does not express response retention (see Fig 6). The patterns of cortical activity with step-like shifts of the location of activity (Fig 8C and 8D) might not be compatible with perceiving apparent motion.

**Fig 9:**
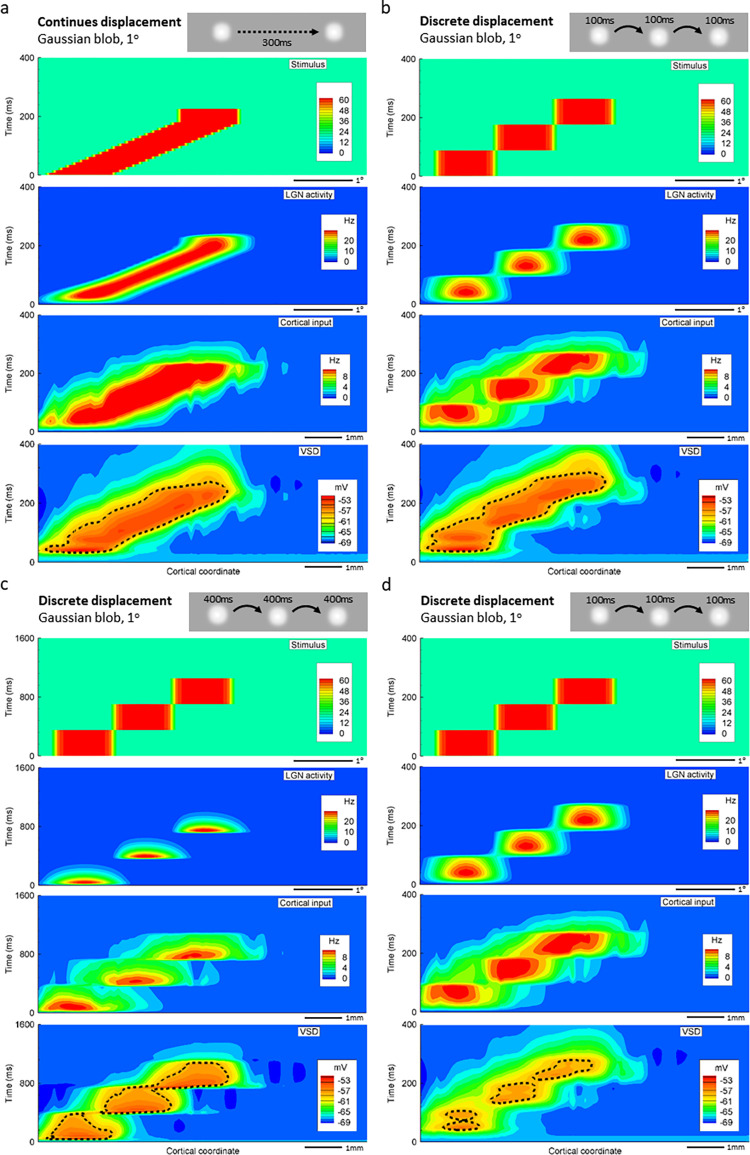
Spatio-temporal profiles of activity evoked in CBRD model by a moving stimulus and patterns of flashing stimuli that may produce apparent motion effect. (a-d) Space-time plots of the stimulus (top panels), firing rate in the LGN (2^nd^ from the top panels), input to the cortex taking into account response kinetics of thalamic neurons, spatial distribution and overlap of thalamic projections to the cortex (3^rd^ from the top panels), and activity in the visual cortex (bottom panels; activity measured as mean membrane potential of cortical neurons, which provides an estimate of the voltage-sensitive dye signals (VSD), black dotted lines show the boundaries of pronounced response activity at the level -58.5mV) evoked in the model by either a moving spot (a) or spot stimuli flashing at three locations (b-d). Data from the same simulation experiments as in Fig 8. Insets show schematic pattern of stimulation. Spatio-temporal pattern of activity produced by a moving stimulus is smooth both in the LGN and visual cortex (a). Flashing stimuli evoke responses at discrete locations in the LGN (b-d, middle panels). In CBRD model with standard settings, spatio-temporal pattern of activity in the visual cortex is smooth when stimuli are flashing at short intervals (b, 100 ms), but consists of discrete areas of activity when stimuli are flashing at long intervals (c, 400 ms; note coarse time scale). In the model with weaker recurrent connections the pattern of cortical activity evoked by rapidly-flashing stimuli (d, 100 ms) also tends to split, with foci at discrete locations.

Fig 9 extends the analysis of activity profiles evoked by the moving or spatially-displaced flashing stimuli, presenting spatio-temporal profiles of the stimulation patterns and activity at different levels in the model. Continuously moving stimulus (Fig 9A, upper panel) evokes activity at continuously and smoothly shifting locations in the LGN (Fig 9A, middle panel). In the cortex, this spatio-temporal pattern of activity with a smooth continuous shift of its focus in both space and time remains (Fig 9A, bottom panel), though the profile of membrane potential changes in the cortex is broader than the profile of firing rate changes in the LGN. Rapid presentation of flashing stimuli at three discrete locations (Fig 9B, upper panel) evokes activity clearly localized at three respective locations in the LGN (Fig 9B, middle panel). In the cortex however, the spatio-temporal pattern of activity becomes different from that in the LGN, with a smooth shift of activity along the three stimulated locations (Fig 9B, bottom panel). For the generation of a smoothly shifting pattern of activity in response to flashing stimuli, both the temporal characteristics of the stimulation and the strength of recurrent connectivity are crucial. With weaker recurrent connections in the cortex, the same stimulus and the same pattern of LGN activity do not produce a smooth spatio-temporal pattern of cortical activity anymore. Rather, the shift of cortical activity between the stimulated locations follows the LGN activity more closely, and becomes step-like but not smooth (Fig 9D, bottom panel). Similarly, spatio-temporal pattern of activity in the cortex expresses clear step-wise shifts between the stimulated locations when the stimuli are presented at lower frequency (400 ms duration) to the model with standard recurrent connectivity (Fig 9C, bottom panel). The difference between activity patterns is markable from comparison of isolines shown by dashed black lines. The isolines in Fig 9A and 9B are simply connected, whereas they are multiply-connected in Fig 9C and 9D. The isolines correspond to one specified level of the VSD signal, -58.5mV. We note, however, that this level of such complex signal as VSD is conditional, because the level at which such representation might be meaningful is hardly known. At least at this level, the patterns are different, and the level is close to the upper limit of the entire range of VSD values, thus can be attributed to the boundary of the high activity zone.

Thus, similar to experimental results, apparent motion stimulation (rapid succession of flashes at three spatially displaced locations) produces in the model with strong recurrent connectivity a profile of cortical activity with a smooth shift of focus that closely resembles the profile produced by a moving stimulus. Reducing the rate of presentation of flashing stimuli, or reducing the strength of recurrent connectivity in the model, disrupts the smooth pattern of cortical activity, making the shifts of activity between stimulus locations step-like.

## Discussion

Apparent motion is an illusion of motion produced by stationary stimuli consecutively flashing at two (or more) spatially-displaced locations. For certain combinations of spatial separation and timing of the stimuli, this configuration is perceived as an object continuously moving between these locations (for review see [13–15]). In the retinotopic representation of space in primary visual cortex, neuronal correlate of apparent motion is a gradual propagation of activity from one location to the other. Indeed, experimental data revealed such spread of activity over the visual cortex, and demonstrated that apparent-motion stimuli produce activity patterns that are closely resemble those evoked by really moving stimuli [4, 16–18].

Here we show that a biologically detailed conductance-based refractory density model (CBRD [22, 24]) of primary visual cortex can reproduce these experimental findings. In the model, apparent-motion stimuli produce profile of activity with a peak that is smoothly shifting from one stimulus location to the other. A smooth shift of activity from one location to the other is produced because response to a flashing stimulus at one location continues for some time after the stimulus is turned off, and therefore temporally overlaps with the beginning of response to the stimulus flashing at the next location. Prolongation of activity beyond the time of stimulus presentation and its gradual fading down on a time scale comparable to the time scale of stimuli presentation are essential for producing a smooth spatio-temporal profile of activity, similar to the profile produced by a continuously moving object.

Our results show that smooth flow of activity between locations representing consecutive flashing stimuli is disrupted when the duration of each stimulus is increased. With such stimulation, transition of activity between stimuli locations becomes step-wise. This can be explained by a decreased prolongation of activity in response to long stimuli, since by the end of a long stimulus the response declines due to adaptation and thus decay of activity after the stimulus offset starts from a lower level. In addition, because of a longer duration of the stimulus, the response prolongation relative to the stimulus duration is proportionally smaller, thus increasing the discrepancy between the time scale of response prolongation and time scale of the stimulus. Notably, such transition from a smooth spread of activity to a step-like shift, observed in our model, has a parallel in perceptual phenomena: substantial increase of the duration of flashing stimuli leads to a shift from perception of apparent movement to perceiving stimuli as disappearing at one location and appearing at the other [4, 15].

Smooth flow of activity in response to apparent-motion stimuli is also disrupted in a model with reduced strength of recurrent connections. In that case, activity decays rapidly after the stimulus offset, leaving only a weak trace by the time of appearance of activity in response to the stimulus presented at the next location. The resulting step-like shift of activity between stimulated locations is not compatible with spatio-temporal patterns of activity measured in the visual cortex during presentation of apparent-motion stimuli [4, 15, 17, 18].

The above considerations stress the role of the prolongation of responses to brief stimuli–**response retention**–for producing in the visual cortex spatio-temporal profiles of activity which are compatible with, and presumably leading to, perception of apparent motion: a smooth, gradual spread of activity from one location to the other.

Therefore, for a mechanistic understanding of this phenomena, it is important to understand the origin of response retention. While response retention is a network phenomenon, because it lasts longer than the longest response of a single isolated neuron, it is still influenced by the properties of individual neurons and synapses composing the network. Response retention was completely abolished in a model with blocked NMDA receptors, which would normally amplify AMPA-mediated excitation by increasing both the amplitude and the duration of single-cell responses [29, 30]. In the modeling study [31], activity of NMDA receptors were found to be important for reproducing the apparent motion-style profiles of activity. At the same time, non-linearity of the voltage-dependence of NMDA receptor activation [32] was not a critical factor in amplification of activity, because elimination of the non-linearity by performing simulations in zero magnesium did not change the response retention. Other cellular and synaptic properties with known effects on neuronal responses influenced response retention in a predictable way. With increased synaptic depression, response retention decreased. With suppressing conductances that normally limit repetitive cell firing (AHP, M-current), response retention becomes stronger.

Critical role in mediating response retention is played by the network properties, in the first place by the recurrent connections. In the ring models, response retention occurs in the same range of the strengths of recurrent connectivity that support the appearance of bump attractor which is invariant to input despite large changes of the input amplitude [20, 33]. Thus, reproducing response retention in the classical ring model does not require additional tuning of parameters: With parameter settings supporting the invariance of the bump attractor and the desired properties of the signal sharpening and orientation tuning, the model also supports response retention. In the models used in this study, both with simple ring structure [19–21] and with retinotopic representation of space, decreasing the strength of recurrent connections resulted in reduced response retention. In the CBRD model with weak connections, reduction of response retention was accompanied by the disruption of the smooth flow of activity between consecutive locations in response to apparent-motion stimuli. Importance of the recurrent connections is stressed by the fact that the effect of the reduction of their strength on response retention could not be compensated by increasing the initial excitation by applying stronger stimuli. In agreement with this observation, the neural field model [34], while omitting orientation processing and biophysical details, was able to reproduce both long-lasting responses to short stimuli and apparent motion-style activity profiles due to fitting the mixing ratio of excitatory to inhibitory signals, which indirectly reflects the contribution of the recurrent connections.

There is an optimal range of recurrent connectivity for mediating response retention. With connectivity too weak, no response retention occurs, and with connectivity too strong, the network becomes hyperexcitable and enters a high-activity state. Mechanisms underlying the effects of the recurrent connectivity strength on network dynamics can be understood by considering changes of the firing rate in excitatory and inhibitory neuronal populations in response to external stimuli. Neuronal networks operate in a balanced excitation/inhibition regime [35–38]. Changing the strength of the recurrent connections change the range of excitatory and inhibitory firing rates in which balanced regime of activity can last for prolonged time periods. When activity is within this range, firing rates change slowly; outside of this range the system is out of balance and firing rates (of excitatory, inhibitory or both populations) tend to change rapidly and bring the system in the range of slow changes and eventually to the equilibrium point. With weak recurrent connections the range of slow rate changes is small. After a perturbation produced by external stimulus, only a small portion of the trajectory of firing rates recovering to the equilibrium has to pass through this slow-change region, and thus activity does not outlast the stimulus much. With increasing the strength of recurrent connections, the region of slow rate changes (corresponding to the range of activity in about balanced excitation/inhibition regime) is expanding. Bigger portion of the trajectory of firing rates goes through this region, and activity after the stimulus offset can last longer. With very strong recurrent connections and further increase of the region of slow rate changes, an additional attractor point of balanced excitatory/inhibitory activity appears at high firing rates. In the high-rate state, the network responds to stimulation with only moderate further increase of activity because of strong background inhibition, which also effectively prevents prolongation of activity and response retention.

Response retention and smooth spatio-temporal profiles of activity in response to apparent motion stimuli are not present in the LGN, but originate in the cortex. Notably, response retention increases as activity propagates through several layers of neurons with recurrent connectivity. We found this consistently, both in simple-structure ring models as well as in biologically detailed CBRD model. An increase of response retention implies that spatio-temporal profiles of activity in response to apparent-motion stimuli might become progressively smoother upon propagation of activity through the layers of cortical network, and thus more and more similar to the profiles produced by moving stimuli. This could be achieved both during propagation of activity through the layers within V1, as well as during propagation between cortical areas. Indeed, imaging neuronal activity in ferret visual cortex revealed a role of motion-dependent feedback connections to areas 17/18 from areas 19 and 21 in generating patterns of smoothly propagating activity between the two locations of apparent-motion stimuli [5]. Interaction with extrastriate visual areas during perception of apparent motion is also suggested by results of fMRI studies in humans [17, 18].

In the present study we focused on the response retention, which is an essential feature for producing smooth spatio-temporal profiles of activity in response to apparent-motion stimuli, and on the role of recurrent connectivity in mediating response retention. Obviously, however, that other features and mechanisms operating in neuronal networks also play important roles in shaping spatio-temporal profiles of activity. One such feature is a point-spread function, characterized by the spread of activity in response to a local stimulation. Point-spread function consists of a region of activated neurons surrounded by a broader area of subthreshold activity [4, 28, 39, 40], determined by the extent of local and long-range horizontal connections. In turn, this determines the range of spatial integration, and for the case of apparent motion maximal distance at which two sequentially flashing stationary stimuli could be perceived as one moving stimulus. Investigation of how details of horizontal connectivity affect responses to apparent-motion stimuli was outside the scope of the present study, therefore we did not vary the respective parameters, but used the values inherited from previous models [20–22]. Further mechanisms which are intrinsic for neuronal networks and were inherited in the CBRD model used in this study from previous work on modeling orientation and direction selectivity of V1 neurons [21], include e.g. differential spatial extension of excitatory and inhibitory connections, with wide-range excitation and local inhibition, creating a complex balance in their interaction and strong nonlinearity in responses to moving stimuli, as well as detail of modeling basic electrophysiology of neurons, such as the use of conductance-based approach, taking into account shunting interactions between synaptic inputs. Earlier studies stressed the importance of these features for reproducing spatio-temporal dynamics of responses to apparent-motion stimuli in visual cortex models [16, 41]. The authors of the recent study [16] specifically stress the importance of shunting interactions and differential gain of excitatory and inhibitory neurons for reproducing suppressive waves produced by each of the stationary stimuli in the apparent-motion stimulus configuration. Such suppressing waves help to restrict activity spread and reduce the ambiguity of interpretation of V1 activity by readout structures, helping to solve the correspondence problem. In contrast to our study and the neural field model from [31], other modeling studies [16, 31] emphasized the importance of long-range excitatory intracortical connections and significant dispersion of axonal delays as the underlying factors for the propagation of wave-like activity. The wide spread of subthreshold postsynaptic activity results from excitation in the domain retinotopically corresponding to a stimulus; this feedforward intracortical spread to periphery being the mechanism for the perception of apparent motion. A comparison between our short recurrent connection-based mechanism and their long feedforward mechanism needs to be conducted in future modeling studies which also relate to mechanisms of divisive normalization wave and balance of short-versus-long-range interactions [42]. Another aspect concerns the orientation of continuous streaks emerging in response to non-oriented spot-like stimulus in experiments [43] and observed in our simulations as asymmetry of responses. Presumably, this effect is caused by elongation of neuronal receptive fields and more precisely by the elongation of the thalamocortical connection footprint according to the orientation map, and further affected by intracortical interactions. This phenomenon is to be modelled in future studies. Together, these results show the importance of multiple mechanisms and their interaction in reproducing spatio-temporal patterns of activity in responses to complex stimuli.

To conclude, studies of spatio-temporal patterns of activity in visual cortex allowed to link the perceptual phenomenon of apparent motion to specific patterns of neuronal activity. Modelling approaches help to understand features of elements, wiring of neuronal networks and dynamic interactions of neuronal populations that produce patterns of activity, which are then interpreted as moving objects by the downstream visual areas.

## Supporting information

S1 Methods CBRD-based model. (PDF)
