# Supplementary material for: Response retention and apparent motion effect in visual cortex models
Source: PLoS One. 2023 Nov 2;18(11):e0293725. doi: 10.1371/journal.pone.0293725 (PMC10621977; doi:10.1371/journal.pone.0293725)
Supplement: S1 Methods — (PDF) [file pone.0293725.s001.pdf]

S1 Methods

CBRD-based model

The complex model includes convolution-based description of LGN activity and CBRD-based description of neuronal population interactions in V1.

Model of LGN, and input to V1. Following model that describes activity of orientation- and direction-selective V1 neurons [1], we distinguish different types of LGN neurons that innervate V1 neurons, transient and sustained ones, whose activity is characterized by the firing rates $L^T_{LGN}(x,y,t)$ and $L^S_{LGN}(x,y,t)$, respectively. At any given time moment, the firing rate of an LGN neuron is calculated as a convolution of the stimulus with a receptive field (RF). The firing rate $L^T_{LGN}(x,y,t)$ at any given time moment t is a rectified convolution of the stimulus $S(x,y,t)$ distributed across the retina with a spatial-temporal receptive field (RF) $D(x,y,t)$ [2]. The firing rate is calculated as follows:

$$\tilde{L}_{LGN}(x,y,t) = \int_0^t d\tau\ dx'dy'D(x',y',\tau)\ S(x',y',t-\tau) \quad (1)$$

$$L^T_{LGN}(x,y,t) = [\tilde{L}_{LGN}(x,y,t)]_+, \quad (2)$$

where the receptive field $D(x,y,t)$ is approximated as the difference of central $D^{cen}(x,y,t)$ and surround $D^{sur}(x,y,t)$, each being separable in space and time, and round. The spatial kernel displays a center-surround structure determined by axons from retinal ganglion cells-described as a difference of two axisymmetric Gaussian functions:

$$D(x,y,t) = D^{cen}(x,y,t) - D^{sur}(x,y,t) = \frac{D^{cen}_t(t)-B}{\pi\sigma^2_{cen}} exp\left(-\frac{x^2+y^2}{\sigma^2_{cen}}\right) - \frac{D^{sur}_t(t)-B}{\pi\sigma^2_{sur}} exp\left(-\frac{x^2+y^2}{\sigma^2_{sur}}\right) \quad (3)$$

An example of the LGN neuron's RF is shown in **Fig. 5**. The temporal component is determined by alpha-functions, with a coefficient controlling the balance between early and late components of LGN cell responses.

Here $D^{cen}_t(t)$ and $D^{sur}_t(t)$ are the temporal evolution functions

$$D^{cen}_t(\tau) = t/\tau^2_{cen}\, exp(-t/\tau_{cen}) - t/\tau^2_{late}\, exp(-t/\tau_{late}) \quad (4)$$

and

$$D^{cen}_t(\tau) = t/\tau^2_{sur}\, exp(-t/\tau_{sur}) - t/\tau^2_{late}\, exp(-t/\tau_{late}) \quad (5)$$

with the time constants τ_{cen}, τ_{sur} and τ_{late}, respectively. The spatial scales of the RF are σ_{cen} and σ_{sur}. The parameters were: $\tau_{cen} = 10ms$, $\tau_{sur} = 20ms$, $\tau_{late} = 64ms$, σ_{cen}= 0.3 deg., σ_{sur}= 1.5 deg. The parameters are from [1, 2]. The *sustained cells* have in 3.5 times slower kinetics [3]. The firing rate $L^S_{LGN}(x, y, t)$ is calculated from the Eqs. (1-5) with the time constant $\tau^S_{cen} = 3.5\tau_{cen}$, $\tau^S_{sur} = 3.5\tau_{sur}$, $\tau^S_{late} = 3.5\tau_{late}$ instead of τ_{cen}, τ_{sur} and τ_{late}.

Thalamic input to V1. Orientation and direction selectivity is determined by the footprint of the LGN-to-V1 projections as well as by properties of the LGN neurons. The thalamic input is determined as the firing rate $\phi_{Th\ E}(x, y, t)$, which is a convolution of the LGN neuronal activity of the transient and sustained cells, $L_{LGN}(x, y, t)$ and $L^S_{LGN}(x, y, t)$, respectively, with the footprint function $D^{LGN-V1}(x, y, \tilde{x}, \tilde{y}, i)$, where $i(x, y, \tilde{x}, \tilde{y})$ is the index of the LGN neuronal population, which attributes T or S indexes, respectively:

$$\phi_{Th\ E}(x, y, t) = d\tilde{x}d\tilde{y} \sum_i D^{LGN-V1}(x, y, \tilde{x}, \tilde{y}, i(x, y, \tilde{x}, \tilde{y}))L^{i(x,y,\tilde{x},\tilde{y})}_{LGN}(\tilde{x}, \tilde{y}, t) \quad (6)$$

The footprint of a direction-selective V1 neuron splits into two halves along the axis of elongation, each sending signals from either transient or sustained thalamic cells. The width and elongation of the footprints were 0.3 deg. and 0.8 deg., respectively. The neighboring V1 neurons that belong to different orientational hypercolumns have footprints of similar shapes and prefer the same orientation but opposite directions of the stimulus movement. In the T-S cell based mechanism of direction selectivity, the input firing rate is

$$\phi_{Th\ E}(x, y, t) = d\tilde{x}d\tilde{y}D^{LGN-V1}(x, y, \tilde{x}, \tilde{y})L^{i(x,y,\tilde{x},\tilde{y})}_{LGN}(\tilde{x}, \tilde{y}, t), \quad (7)$$

where L^T_{LGN} is the activity of the LGN neuron $(\tilde{x}, \tilde{y})$; $D^{LGN-V1}(x, y, \tilde{x}, \tilde{y})$ is the LGN-to-V1 footprint with the width across preferred orientation σ_{pref} and the width across orthogonal orientation σ_{orth}; $i(x, y, \tilde{x}, \tilde{y})$ is the index of T or S neurons, whose firing rates are calculated with different time constants in the temporal kernel of the convolution. It is defined according to the footprint:

$$i(x, y, \tilde{x}, \tilde{y}) = "S", \quad if \quad (-1)^{i_{PW}+j_{PW}}x^{'} > 0\,; \quad "T", \ otherwise. \quad (8)$$

$D^{LGN-V1}(x, y, \tilde{x}, \tilde{y})$, x' and y' are given by the following equations:

$$D^{LGN-V1}(x, y, \tilde{x}, \tilde{y}) = \frac{1}{\pi\ \sigma_{pref}\sigma_{orth}} exp\left(-\frac{x^{'2}}{\sigma^2_{pref}} - \frac{y^{'2}}{\sigma^2_{orth}}\right), \quad (9)$$

$$x^{'} = (\tilde{x} - x_{cf})\cos\theta - (\tilde{y} - y_{cf})\sin\theta \quad (10)$$

$$y^{'} = (\tilde{x} - x_{cf})\sin\theta + (\tilde{y} - y_{cf})\cos\theta. \quad (11)$$

Here (x_{cf}, y_{cf}) are the coordinates of the center of the footprint of V1 neuron in LGN, $x_{cf} = x_{cf}(x, y)$, $y_{cf} = y_{cf}(x, y)$.

Model of V1. V1 is modeled as a 2-dimensional continuum of neuronal populations. Each point of the cortical continuum contains two neuronal populations, excitatory (E) and inhibitory (I), connected by AMPA (α-amino-3-hydroxy-5-methyl-4-isoxazolepropionic acid), NMDA (N-methyl-d-aspartate), and GABA-A mediated synapses providing recurrent intracortical interactions and AMPA and NMDA for the geniculate input. The strengths of the external connections correspond to the pinwheel architecture, and thus neurons receive inputs in accordance with their orientation and direction preferences, taken from the experimental map shown in Fig.5. The profile of the intracortical connections is isotropic, i.e., the maximum conductances depend on the distance between the pre- and postsynaptic populations.

We define a neuronal population as a group of similar neurons that receives both a common input from presynaptic populations and an individual noise. The mathematical description of each population is based on the probability density approach, namely, the CBRD approach [4], The model for each *E*- and *I*- neuronal populations takes into account two neuronal compartments and a set of voltage-gated ionic currents, including the adaptation currents.

The neuronal population firing rate determines the presynaptic firing rate, which in turn controls the dynamics of synaptic conductances. The presynaptic firing rate predetermined by the excitatory population firing rate determines the dynamics of AMPA and NMDA synaptic conductances. The inhibitory population controls the GABA conductance. The synaptic conductances are the input signals for the postsynaptic neuronal populations. The membrane voltage distribution across t^* determines the output firing rate and so on.

Model of a single neuronal population. In the CBRD model, the membrane potentials and ionic channel states of the neurons of one population are dispersed due to the noise, and thus they are distributed in a space of neuronal refractority. The refractority state is characterized with the time elapsed since the last spike, t^*. Single population dynamics are governed by the equations for neuronal density, the mean over noise realizations voltage, and the gating variables. Neurons that fire contribute to the population firing rate, which is the output measure of the population activity.

The firing rate can be quite precisely and computationally effectively calculated by solving a system of equations in partial derivatives, 1-d transport equations. The equations govern the evolution of

neuronal states in the t^* - phase space. They contain the Hodgkin-Huxley equations for the membrane voltage and gating variables, parameterized by t^*, as well as the equation for the neuronal density in t^*-space, $\rho^j(t,t^*)$. The output characteristic of the population's activity is the firing rate $\nu^j(t)$, which is equal to ρ^j in the state of a spike, $t^* = 0$. The equations written below describe an excitatory population of adaptive regular spiking pyramidal cells according to [4] and [5].

Basic neurons have 2-compartments with the somatic and dendritic voltages $U^j(t,t^*)$ and $U_d^j(t,t^*)$. In comparison with one-compartment model, the extra parameters is the ratio of dendritic to somatic conductances γ and the dendritic length. We assume that the inhibitory synapses are located at soma, contributing into the somatic synaptic current I_{soma}, whereas the excitatory synapses are at dendrites, determining the dendritic synaptic current I_{dendr}. Parameterized by t^*, the governing equations for the population j (E or I) are as follows:

$$\frac{\partial \rho^j}{\partial t} + \frac{\partial \rho^j}{\partial t^*} = -\rho^j H(U^j, g_{tot}^j), \qquad (12)$$

$$C\left(\frac{\partial U^j}{\partial t} + \frac{\partial U^j}{\partial t^*}\right) = -g_L(U^j - V_{rest}) + \frac{2\gamma}{l}\, g_L(U_d^j - U^j)$$

$$+I_{voltage-gated}^j(U^j, t, t^*) + I_{soma} \qquad (13)$$

$$C\left(\frac{\partial U_d^j}{\partial t} + \frac{\partial U_d^j}{\partial t^*}\right) = -g_L(U_d^j - V_{rest}) - \frac{2}{l}\, g_L(U_d^j - U^j) + \frac{I_{dendr}}{\gamma}, \qquad (14)$$

where $g_{tot}^j(t,t^*)$ is the total conductance; l is the square ratio of the dendritic length to the characteristic length. The somatic and dendritic synaptic currents I_{soma} and I_{dendr} are calculated as

$$I_{soma} = g_{GABA,j}(t)(V_{GABA} - U^j)$$

$$I_{dendr} = \left(\frac{l\,\tau_m^0}{2}\frac{d}{dt} + 1 + \frac{l}{2}\right)\left(g_{AMPA,E}(t)(V_{AMPA} - U^j) + g_{NMDA,E}(t)(V_{NMDA} - U^j)\right),$$

where the differential operator represents the solution of the reverse problem of dendritic current estimation from somatically registered-like conductances [6]. We imply that the synaptic conductance kinetics is estimated from somatic responses to stimulation of presynaptic neuronal population, thus it implicitly accounts not only the kinetics of synaptic channels but also the dendritic and axonal propagation delays. For the dendritic compartment, the differential operator sharpens the transient effect of the channels, thus providing better agreement between somatic postsynaptic currents and potentials. Note that we take into account this sharpening only for glutamatergic channels by placing them on the dendritic compartment [7].

Hazard function. The source term in the eq.(1) is the hazard function H which is defined as the probability for a single neuron to generate a spike, if known actual neuron state variables. The hazard function H has been approximated in [5] for the case of color noise as a function of $U^j\,(t)$ and$g_{tot}^{j}(t,t^*)$,, and the noise amplitude in the resting state σ_V^0, the spike threshold voltage V_{th} and the ratio of membrane to noise time constants $k = \tau_m/\tau_{Noise}$:

$$H(U) = A + B, \tag{15}$$

$$A = \frac{1}{\tau_m} e^{0.0061-1.12\,T-0.257\,T^2-0.072\,T^3-0.0117\,T^4}\left(1-(1+k)^{-0.71+0.0825(T+3)}\right),$$

$$B = \sqrt{2}\,\left[-\frac{dT}{dt}\right]_+ \sqrt{\frac{2}{\pi}}\,\frac{exp(-T^2)}{1+erf(T)},\;\; T = \frac{V_{th}-U^p}{\sqrt{2}\,\sigma_V}\sqrt{\frac{g_{tot}^p}{g_{tot}^0}},$$

where T is the membrane potential relative to the threshold, scaled by noise amplitude; A is the hazard for a neuron to cross the threshold because of noise, derived analytically and approximated by exponential and polynomial for convenience; B is the hazard for a neuron to fire because of depolarization due to deterministic drive, i.e. the hazard due to drift in the voltage phase space. Note that the H -function is independent of the basic neuron model and does not contain any free parameters or functions for fitting to any particular case. Thus, H -function is the same for excitatory and inhibitory populations.

Voltage-dependent channels of excitatory neurons. The ionic currents $I_{voltage-gated}^{E}(U^E,t,t^*) = -I_{DR} - I_A - I_M - I_{AHP}$ include the voltage-dependent potassium currents I_{DR} and I_A responsible for spike repolarization, the slow potassium current I_M that contributes to spike frequency adaptation, the cation current I_H and the potassium current I_{AHP}, implicitly dependent on calcium dynamics, which also contributes to spike frequency adaptation. The approximating formulas for the currents I_{Na}, I_{DR}, I_A, I_M and I_H are taken from [8]; the approximation for I_{AHP} is from [9].

The voltage-dependent potassium current I_{DR}:

$$I_{DR}(U^E,t,t^*) = \overline{g}_{DR}\,x(t)\,y(t)\,(U^E(t) - V_{DR}), \tag{16}$$

$$\frac{\partial x}{\partial t} + \frac{\partial x}{\partial t^*} = \frac{x_\infty(U^E)-x}{\tau_x(U^E)}, \tag{17}$$

$$\frac{\partial y}{\partial t} + \frac{\partial y}{\partial t^*} = \frac{y_\infty(U^E)-y}{\tau_y(U^E)} \tag{18}$$

$$\tau_x = 1/(a+b) + 0.8\,ms;$$

$$x_\infty = a/(a+b),$$

$$a = 0.17\,exp(\,(U^E+5)\cdot 0.090)\,ms^{-1},$$

$$b = 0.17\,exp(-(U^E+5)\cdot 0.022)\,ms^{-1},$$

$$\tau_y = 300\,ms,$$

$$y_\infty = 1/(1+exp(\,(U^E+68)\cdot 0.038));$$

The voltage-dependent potassium current I_A:

$$I_A(U^E,t,t^*) = \overline{g}_A\, x^4(t)\, y^3(t)\, (U^E(t) - V_A), \tag{19}$$

$$\frac{\partial x}{\partial t} + \frac{\partial x}{\partial t^*} = \frac{x_\infty(U^E) - x}{\tau_x(U^E)}, \tag{20}$$

$$\frac{\partial y}{\partial t} + \frac{\partial y}{\partial t^*} = \frac{y_\infty(U^E) - y}{\tau_y(U^E)} \tag{21}$$

$$\tau_x = 1/(a_x+b_x) + 1\,ms;$$

$$x_\infty = a_x/(a_x+b_x),$$

$$a_x = 0.08\,exp(\,(U^E+41)\cdot 0.089)\,ms^{-1},$$

$$b_x = 0.08\,exp(-(U^E+41)\cdot 0.016)\,ms^{-1},$$

$$\tau_y = 1/(a_y+b_y) + 2\,ms;$$

$$y_\infty = a_y/(a_y+b_y),$$

$$a_y = 0.04\cdot exp(-(U^E+49)\cdot 0.11)\,ms^{-1},$$

$$b_y = 0.04\,ms^{-1};$$

The voltage-dependent potassium current I_M:

$$I_M(U^E,t,t^*) = \overline{g}_M\, x^2(t)\, (U^E(t) - V_M), \tag{22}$$

$$\frac{\partial x}{\partial t}+\frac{\partial x}{\partial t^*}=\frac{x_\infty(U^E)-x}{\tau_x(U^E)}, \quad (23)$$

$$\tau_x = 1/(a+b)+8\,ms,$$

$$x_\infty = a/(a+b),$$

$$a = 0.003\,exp(\,(U^E+45)\cdot 0.135)\,ms^{-1},$$

$$b = 0.003\,exp(\,-\,(U^E+45)\cdot 0.090)\,ms^{-1};$$

The adaptation current I_{AHP}:

$$I_{AHP}(U^E,t,t^*) = \overline{g}_{AHP}\,x(t)y(t)\,(U^E(t)-V_K), \quad (24)$$

$$\frac{\partial x}{\partial t}+\frac{\partial x}{\partial t^*}=\frac{x_\infty(U^E)-x}{\tau_w(U^E)}, \quad (25)$$

$$\frac{\partial y}{\partial t}+\frac{\partial y}{\partial t^*}=\frac{y_\infty(U^E)-y}{\tau_y(U^E)} \quad (26)$$

$$\tau_w = 2000/(3.3\,exp(\,(U^E+35)/20)+exp(\,-\,(U^E+35)/20))\,ms,$$

$$x_\infty = 1/(1+exp(\,-\,(U^E+35)/4)),$$

$$\tau_y = 1000\,ms,$$

$$y_\infty = 1/(1+exp(\,(U^E+40)/5))\,;$$

Voltage-dependent channels of interneurons. The model of the fast-spiking single-compartment interneurons taken from [10] reduces the voltage-gated current to an only potassium current

$$I^I_{voltage-gated}(U^I,t,t^*) = \overline{g}_K\,n^4(t)\,(U^I(t)-V_K), \quad (27)$$

$$\frac{\partial n}{\partial t}+\frac{\partial n}{\partial t^*}=\frac{n_\infty(U^I)-n}{\tau_n(U^I)}, \quad (28)$$

$$\tau_n = (0.5+2/(1+exp(\,0.045(U^I-50)ms;$$

$$n_\infty = 1/(1+exp(\,-\,0.045(U^I+10))).$$

Boundary conditions. According to the conservation of the number of neurons in a population, the firing rate is calculated as a sink of neurons from their state t^* due to spiking, $\rho^j(t,t^*)\,H(U^j(t,t^*)$, integrated over the whole phase space, i.e.

$$\nu^j(t) \equiv \rho^j(t,0) = \int_{+0}^{\infty} \rho^j(t,t^*)\, H(U^j(t,t^*))dt^*. \tag{29}$$

It is a boundary condition for eq.(12).

The spike duration is taken into account by introducing the time interval $0 < t^* < \Delta t_{AP}$ during which the voltage and the gating variables are fixed to their reset values. It defines the boundary conditions at $t^* = \Delta t_{AP}$ which are as follows:

$$U^E(t, \Delta t_{AP}) = V_{reset}, \tag{30}$$

$$U_d^E(t, \Delta t_{AP}) = V_{rest}; \tag{31}$$

$$I_{DR}:\ x(t, \Delta t_{AP}) = 0.262,\ y(t, \Delta t_{AP}) = 0.473; \tag{32}$$

$$I_A:\ x(t, \Delta t_{AP}) = 0.743,\ y(t, \Delta t_{AP}) = 0.691. \tag{33}$$

The reset values for the fast gating variables in eqs.(32, 33) were obtained with the basic single neuron model. With a rather arbitrary input providing a spike, these values were measured at the moment of a voltage maximum at the spike. The reset level for each slow conductance in the CBRD model was calculated as a sum of its value at a peak of spike-release distribution in the t^*-space and an increment at spike:

$$I_M:\ x(t, \Delta t_{AP}) = x(t, t^{*p}) + 0.175\,(1 - x(t, t^{*p})); \tag{34}$$

$$I_{AHP}:\ w(t, \Delta t_{AP}) = w(t, t^{*p}) + 0.018\,(1 - w(t, t^{*p})); \tag{35}$$

where t^{*p} is such that

$$\rho(t, t^{*p})\, H(t, t^{*p}) = \max_{0<t^*<+\infty} \rho(t, t^*)\, H(t, t^*).$$

The increment values for the slow gating variables in eqs.(34-35) were also measured at a single spike of the single neuron model.

Parameters for excitatory neurons are as follows:

$$V_{DR} = -70\ mV,\ V_A = -70\ mV,\ V_M = -80\ mV,\ V_{AHP} = -70\ mV,$$

$$\overline{g}_{DR} = 0.76\ \mu S/cm^2,\ \overline{g}_A = 4.36\ \mu S/cm^2,$$

$\overline{g}_M = 0.4\,\mu S/cm^2,\ \overline{g}_{AHP} = 0.3\,\mu S/cm^2,$

$\tau_m^0 = C/g_{tot}^0 = 14.4\,ms,\ (g_L = 0.034\,\mu S/cm^2),$

$V_{rest} = -65\,mV,\ \ V_{th}(t^*) = (-50 + 50\,exp(-t^*/10\,ms))\,mV,$

$V_{reset} = -40\,mV,\ \ \Delta t_{AP} = 1.5\,ms,$

$\gamma = 2.85,\ \ C = 0.7\,\mu F/cm^2,\ \ \sigma_V = 6(1 + g_{syn}/g_{tot}^0)\,mV,$

$S = 0.7 \cdot 10^{-5}\,cm^2$

Here g_{tot}^0 is the total somatic conductance at rest, and g_{syn} is the total synaptic conductance; S is the membrane area. The dependence of $V_{th}(t^*)$ is taken from a full single neuron model [4], allowing to take into account the effect of sodium channel inactivation on the threshold dynamics. σ_V is the noise amplitude meaning the dispersion of individual neuron's voltage fluctuations in a stationary state. Its scaling with g_{syn} approximately reflects the fact of the synaptic noise increase with the increase of mean synaptic drive.

For interneurons:

$V_K = -80\,mV,\ \ \overline{g}_K = 40\,mS/cm^2,$

$g_L = 0.1\,\mu S/cm^2,\ \ (\tau_m^0 = C/g_{tot}^0 = 9.6\,ms),$

$V_{rest} = -65\,mV,\ \ V_{reset} = -40\,mV,$

$V_{th}(t^*) = (-50 + 20\,exp(-t^*/10\,ms)\,mV,$

$\Delta t_{AP} = 1.4ms,\ \ \gamma = 2.85,\ \ C = 1\,\mu F/cm^2,$

$\sigma_V = 6(1 + g_{syn}/g_{tot}^0)\,mV,\ \ S = 4.5 \cdot 10^{-5}\,cm^2$

When calculating the dynamics of a neural population, the integration of eqs.(12-26) determines the evolution of the distribution of voltage U^E across t^*. Then, the effect of crossing the threshold and the diffusion due to noise are taken into account by H -function, eq.(19), substituted into the equation for neuronal density (12). The integral (29) results in the output firing rate $\nu^E(t)$.

Representative neurons

Representative neurons of each of the populations were modeled according with the basic single neuron model with the same synaptic inputs as for the populations. The activity of the representative neurons does not affect the network. The representative neuron of *E*-population, for instance, is described by the equations for the membrane voltage, eq. (13,14), where the sum of partial derivatives were substituted by the total derivative in time *t,* and the sodium current was explicitly present in the right-hand part of Eq. (13). The sodium current dependent on voltage *U* was approximated by the 4-state Markov model [8]:

$$I_{Na}(t) = \overline{g}_{Na}\, x_1(t)(U(t) - V_{Na}),$$

$$x_1 + x_2 + x_3 + x_4 = 1,$$

$$\frac{dx_i}{dt} = \sum_{j=0,j\neq i}^{4} A_{j,i}x_j - x_i \sum_{j=0,j\neq i}^{4} A_{i,j},\ \ i = 1,2,3$$

$$A_{1,2} = 3\ ms^{-1},\ A_{1,3} = f_1^{1,3}(U),\ A_{1,4} = f_1^{1,4}(U),$$

$$A_{2,1} = 0,\ A_{2,3} = f_2^{2,3}(U),\ A_{2,4} = 0,$$

$$A_{3,1} = f_1^{3,1}(U),\ A_{3,2} = 0,\ A_{3,4} = f_2^{3,4}(U),$$

$$A_{4,1} = f_1^{4,1}(U),\ A_{4,2} = 0,\ A_{4,3} = 0$$

$$f_1^{i,j}(U) = \{\tau_{min}^{i,j} + 1/\,exp(\,U - V_{1/2}^{i,j} k^{i,j})\}^{-1},$$

$$f_2^{i,j}(U) = \{\tau_{min}^{i,j} + [(\tau_{max}^{i,j} - \tau_{min}^{i,j})^{-1} + exp(\,U - V_{1/2}^{i,j} k^{i,j})]^{-1}\}^{-1},$$

$$\tau_{min}^{1,3} = 1/3\ ms,\ V_{1/2}^{1,3} = -51\ mV,\ k^{1,3} = -2\ mV,$$

$$\tau_{min}^{1,4} = 1/3\ ms,\ V_{1/2}^{1,4} = -57\ mV,\ k^{1,4} = -2\ mV,$$

$$\tau_{min}^{2,3} = 1\ ms,\ V_{1/2}^{2,3} = -53\ mV,\ k^{2,3} = -1\ mV,\ \tau_{max}^{2,3} = 100\ ms,$$

$$\tau_{min}^{3,1} = 1/3\ ms,\ V_{1/2}^{3,1} = -42\ mV,\ k^{3,1} = 1\ mV,$$

$$\tau_{min}^{3,4} = 1\ ms,\ V_{1/2}^{3,4} = -60\ mV,\ k^{3,4} = -1\ mV,\ \tau_{max}^{3,4} = 100\ ms,$$

$$\tau_{min}^{4,1} = 1/3\ ms,\ V_{1/2}^{4,1} = -51\ mV,\ k^{4,1} = 1\ mV.$$

Lognormal distribution of synaptic weights within each population. In order to introduce realistic, lognormal distribution of synaptic weights within a population j (E or I), the CBRD-approach has been generalized [11]. In this case, instead of equal total synaptic current, neurons receive lognormally distributed current. For the current scaled by its mean across the distribution, x, the distribution is

$$\psi(x) = \frac{exp(-(ln\,x)^2/(2\,\sigma_{LN}^2))}{\sqrt{2\pi}\,\sigma_{LN}\,x} \tag{36}$$

The membrane potential of neurons parameterized with x, U_x^j, can be found as

$$U_x^j(t,t^*) = (U^j(t,t^*) - U_{free}^j(t^*))\,x + U_{free}^j(t^*), \tag{37}$$

where $U_{free}^j(t^*)$ is the unperturbed potential defined for zero synaptic input.

The density of neurons parameterized by x and distributed in the phase space t^* is denoted as $\rho_x^j(t,t^*)$. Calculation of $\rho_x^j(t,t^*)$ requires solving of a continuum of eqs.(1) (or eq.(18)) for ρ_x^j instead of ρ^j with $H(U_x^j, dU_x^j/dt)$. The output firing rate is defined as

$$\nu^j(t) = \int_0^\infty \rho_x^j(t,0)\,\psi(x)\,dx \tag{38}$$

In numerical simulations, we set the parameter of the lognormal distribution $\sigma_{LN} = 0.5$ and discretized the x -space by 10 intervals.

Synaptic connections. The types of synapses are denoted by the types of mediator and postsynaptic neurons as follows: $(AMPA, j)$, $(GABA, j)$ and $(NMDA, j)$ with the postsynaptic index j =*E* or*I*.

The kinetics of the synaptic conductances is described following [7] with a second order ordinary differential equation, where the input is the presynaptic firing rate, i.e. as follows

$$g_{AMPA\prime,j}(t) = \overline{g}_{AMPA\prime,j}\,m_{AMPA\prime,j}(t)\,,\ \ g_{AMPA,j}(t) = \overline{g}_{AMPA,j}\,m_{AMPA,j}(t)\,, \tag{39}$$

$$g_{NMDA,j}(t,U^j) = \overline{g}_{NMDA,j}\,f_{NMDA}(U^j(t))\,m_{NMDA,j}(t), \tag{40}$$

$$f_{NMDA}(U^j) = 1/(1 + Mg/3.57\,exp(-0.062\,U^j)),$$

$$g_{GABA,j}(t) = \overline{g}_{GABA,j}\,m_{GABA,j}(t), \tag{41}$$

Mg is the magnesium (Mg2 +) concentration in mM; $m_{s,p}(t)$ is the non-dimensional synaptic conductance which is approximated by the second order ordinary differential equation:

$$\left(\tau_r^{s,j}\tau_d^{s,j}\frac{d^2}{dt^2} + (\tau_r^{s,j} + \tau_d^{s,j})\frac{d}{dt} + 1\right) m_{s,j}(t) = \tau^{s,j}\big(1 - m_{s,j}(t)\big)\phi_{i,j}(t), \quad (42)$$

$$\tau^{s,j} = \left(\tau_r^{s,j} - \tau_d^{s,j}\right) / \left((\tau_d^{s,j}/\tau_r^{s,j})^{\tau_d^{s,j}/(\tau_r^{s,j}-\tau_d^{s,j})} - (\tau_d^{s,j}/\tau_r^{s,j})^{\tau_r^{s,j}/(\tau_r^{s,j}-\tau_d^{s,j})} \right), \quad (43)$$

$$if\ \tau_r^{s,j} \neq \tau_d^{s,j},$$

$$\tau_r^{s,j} e,\ otherwise.$$

Here $\phi_{i,j}$ is the presynaptic firing rate determined by axons of the population i on the postsynaptic population j. In neglect of spatial propagation and temporal delays the presynaptic firing rate is equivalent to the somatic firing rate, i.e. $\phi_{i,j} \equiv \nu_i$. The index s is the synapse type, $s = AMPA$, $GABA$ or $NMDA$; the index $i = Th$ means thalamic input for $s = AMPA'$; $i = E$ for $s = AMPA$ or $NMDA$; and $i = I$ for $s = GABA$; $\overline{g}_{s,j}$ is the maximum conductance, $\tau_r^{s,j}$ and $\tau_d^{s,j}$ are the rise and decay time constants. We imply that the synaptic time constants are estimated from the somatic responses to the stimulation of a presynaptic neuronal population, thus these time constants characterize not only synaptic channel kinetics but the dendritic and axonal propagation delays as well. The time scale $\tau^{s,j}$ is chosen in the form of Eq. (43) in order to provide independence of the maximum of $g_{s,j}(t)$ on $\tau_r^{s,j}$ and $\tau_d^{s,j}$, when $g_{s,j}(t)$ is evoked by a short pulse of $\phi_{i,j}(t)$.

The parameter values were as follows: $\overline{g}_{AMPA',I} = 0$, $\overline{g}_{AMPA',E} = \overline{g}_{AMPA,E} = \overline{g}_{AMPA,I} = 0.4$mS/cm^2, $\overline{g}_{NMDA,E} = \overline{g}_{NMDA,I} = 1.6$mS/cm^2, $\overline{g}_{GABA,E} = 1.2$mS/cm^2, $\overline{g}_{GABA,I} = 0.2$mS/cm^2, $V_{AMPA} = V_{NMDA} = 0$, $V_{GABA} = -77$mV, $Mg = 2$ mM, $\tau_r^{AMPA,E} = \tau_r^{AMPA,I} = 1.7$ ms, $\tau_d^{AMPA,E} = \tau_d^{AMPA,I} = 8.3$ ms, $\tau_r^{NMDA,E} = \tau_r^{NMDA,I} = 6.7$ ms, $\tau_d^{NMDA,E} = \tau_d^{NMDA,I} = 100$ ms, $\tau_r^{GABA,E} = \tau_r^{GABA,I} = 0.5$ ms, $\tau_d^{GABA,E} = \tau_d^{GABA,I} = 20$ ms.

The synaptic depression was taken into account only in simulation for Fig.7a. The factor $x_{i,j}^D$ has been included in the expressions for excitatory conductances (39) and (40). It was modeled with the Tsodyks-Markram model [12]:

$$\frac{dx_{i,j}^D}{dt} = \frac{(1 - x_{i,j}^D)}{\tau_D} - U_D\, x_{i,j}^D\,(t)\,\phi_{i,j}(t),$$

with $\tau_D = 500$ms, $U_D = 0.2$.

Spatial connections. The horisontal corticalconnectivity includes only local isotropic connections. They are defined by the relations for the presynaptic rates $\phi_{i,j}(t)$ dependent on the somatic rates ν_i, where presynaptic and postsynaptic populations are indexed by *i* and *j,* correspondingly. In the case

of 2-d geometry, all variables depend on the spatial coordinates *x,y* along the surface of the cortex. For local isotropic connections we consider a gaussian profile of the strengths of the connections:

$$\phi_{i,j}(t,x,y) = \nu^i(t,x',y')e^{-((x-x')^2+(y-y')^2)/d_{i,j}^2}\,dx'dy'/\,e^{-((x-x')^2+(y-y')^2)/d_{i,j}^2}\,dx'dy' \quad (44)$$

where $d_{i,j}$ is the characteristic length.

The parameters were as follows: $d_{E,E} = 100\mu m$, $d_{E,I} = 500\mu m$, $d_{I,E} = 200\mu m$ and $d_{I,I} = 100\mu m$. Cortex region was 3.32 mm × 0,9 mm, spatial grid 83 × 23, time step 0.1ms.

The software code written in Delphi and the compiled program ``Brain” are available from http://www.ioffe.ru/CompPhysLab/MyPrograms/Brain/Brain.zip.

Quantification of the retention effect. Analyzing the profiles of neural activity in response to a flash stimulus, we estimate the following index C_{loc} as the ratio of the firing rates at the plateau and at the peak of the response:

$$C_{loc} = \frac{r(t^{plateau})}{r(t^{peak})}, \quad (45)$$

where $r(t) = \max_i r_i\,(t)$ is the maximal firing-rate of all neurons. The probe time moment for the plateau was chosen to be 100 ms after the peak $t^{plateau} = 100ms$. For most of the numerical experiments in case of single layer models, the peak was observed at the end of the stimulus.

REFERENCES (Supporting information)

1. Chizhov A, Merkulyeva N (2020). Refractory density model of cortical direction selectivity: Lagged-nonlagged, transient-sustained, and On-Off thalamic neuron-based mechanisms and intracortical amplification. PLOS Computational Biology 16(10): e1008333. https://doi.org/10.1371/journal.pcbi.1008333
2. Dayan P, Abbott LF. Theoretical neuroscience: computational and mathematical modeling of neural systems. The MIT Press. Cambridge, Massachusetts. London, England. 2001.
3. Lien AD, Scanziani M. Cortical direction selectivity emerges at convergence of thalamic synapses. Nature. 2018; 558(7708):80-86. DOI: 10.1038/s41586-018-0148-5.
4. Chizhov AV, Graham LJ (2007) Population model of hippocampal pyramidal neurons linking a refractory density approach to conductance-based neurons. Physical Review E. doi: 10.1103/physreve.75.011924
5. Chizhov AV, Graham LJ (2008) Efficient evaluation of neuron populations receiving colored-noise current based on a refractory density method. Physical Review E. doi: 10.1103/physreve.77.011910

6. Chizhov AV, Sanchez-Aguilera A, Rodrigues S, de la Prida LM (2015) Simplest relationship between local field potential and intracellular signals in layered neural tissue. Physical Review E. doi: 10.1103/physreve.92.062704
7. Chizhov AV (2013) Conductance-based refractory density model of primary visual cortex. Journal of Computational Neuroscience 36:297-319. doi: 10.1007/s10827-013-0473-5
8. Borg-Graham LJ. Interpretations of Data and Mechanisms for Hippocampal Pyramidal Cell Models. In: Cerebral Cortex. Springer Science and Business Media; 1999. pp. 19-138.
9. Whittington MA, Traub RD, Kopell N, and others (2000) Inhibition-based rhythms: experimental and mathematical observations on network dynamics. International Journal of Psychophysiology 38:315-336. doi: 10.1016/s0167-8760(00)00173-2
10. White JA, Chow CC, Ritt J, Soto-Trevino C, Kopell N. Synchronization and oscillatory dynamics in heterogeneous, mutually inhibited neurons. J. Comp. Neuroscience 1998; 5: 5–16.
11. Chizhov AV. Conductance-Based Refractory Density Approach: Comparison with Experimental Data and Generalization to Lognormal Distribution of Input Current. Biol. Cybernetics, 111(5-6):353-364 2017. https://doi.org/10.1007/S00422-017-0727-9
12. Loebel A, Tsodyks M. Computation by ensemble synchronization in recurrent networks with synaptic depression. J Comp Neuroscience. 2002;13: 111-124.
